# *HCN2*‐Associated Neurodevelopmental Disorders: Data from Patients and *Xenopus* Cell Models

**DOI:** 10.1002/ana.27277

**Published:** 2025-06-05

**Authors:** Clara Houdayer, A. Marie Phillips, Marie Chabbert, Jennifer Bourreau, Reza Maroofian, Henry Houlden, Kay Richards, Nebal Waill Saadi, Eliška Dad'ová, Patrick Van Bogaert, Mailys Rupin, Boris Keren, Perrine Charles, Thomas Smol, Audrey Riquet, Lynn Pais, Anne O'Donnell‐Luria, Grace E. VanNoy, Allan Bayat, Rikke S Møller, Kern Olofsson, Rami Abou Jamra, Steffen Syrbe, Majed Dasouki, Laurie H. Seaver, Jennifer A. Sullivan, Vandana Shashi, Fowzan S. Alkuraya, Alexis F. Poss, J. Edward Spence, Rhonda E. Schnur, Ian C. Forster, Chaseley E. Mckenzie, Cas Simons, Min Wang, Penny Snell, Kavitha Kothur, Michael Buckley, Tony Roscioli, Noha Elserafy, Benjamin Dauriat, Vincent Procaccio, Daniel Henrion, Guy Lenaers, Estelle Colin, Nienke E. Verbeek, Koen L. Van Gassen, Claire Legendre, Dominique Bonneau, Christopher A. Reid, Katherine B. Howell, Alban Ziegler, Christian Legros

**Affiliations:** ^1^ Department of Medical Genetics Angers University Hospital Angers France; ^2^ Florey Institute of Neuroscience and Mental Health The University of Melbourne Parkville Victoria Australia; ^3^ School of Biosciences The University of Melbourne Parkville Victoria Australia; ^4^ University of Angers, INSERM, CNRS, MITOVASC, Equipe CarMe, SFR ICAT Angers France; ^5^ Department of Neuromuscular Disorders, UCL Queen Square Institute of Neurology University College London London UK; ^6^ College of Medicine, University of Baghdad Baghdad Iraq; ^7^ Children's Welfare Teaching Hospital Baghdad Iraq; ^8^ Department of Pediatric Neurology Angers University Hospital Angers France; ^9^ Department of Genetics Pitié‐Salpêtrière Hospital, Assistance Publique‐Hôpitaux de Paris, Sorbonne University Paris France; ^10^ University of Lille, CHU Lille, ULR7364 – RADEME, Institute of Medical Genetics Lille France; ^11^ Department of Pediatric Neurology Saint Vincent de Paul Hospital, GHICL Lille France; ^12^ Program in Medical and Population Genetics Broad Institute of MIT and Harvard Cambridge MA USA; ^13^ Division of Genetics and Genomics Boston Children's Hospital Boston MA USA; ^14^ Department for Genetics and Personalized Medicine Danish Epilepsy Centre Dianalund Denmark; ^15^ Department Regional Health Research University of Southern Denmark Odense Denmark; ^16^ Department of Paediatrics Danish Epilepsy Centre Filadelfia Dianalund Denmark; ^17^ Institute of Human Genetics University of Leipzig Medical Center Leipzig Germany; ^18^ Division of Paediatric Epileptology Centre for Paediatrics and Adolescent Medicine, University Hospital Heidelberg Heidelberg Germany; ^19^ Department of Medical Genetics Genomics and Personalized Health at AdventHealth‐Orlando Orlando FL USA; ^20^ Division of Medical Genetics Corewell Health Helen DeVos Children's Hospital Grand Rapids MI USA; ^21^ Department of Pediatrics and Human Development Michigan State University College of Human Medicine Grand Rapids MI USA; ^22^ Department of Pediatrics ‐ Medical Genetics Duke University Durham NC USA; ^23^ Department of Translational Genomics Center for Genomic Medicine, King Faisal Specialist Hospital & Research Center Riyadh Saudi Arabia; ^24^ Pediatrics‐Clinical Genetics and Metabolism School of Medicine, University of Colorado‐Anschutz Medical Campus Aurora CO USA; ^25^ GeneDx Gaithersburg MD USA; ^26^ Murdoch Children's Research Institute Melbourne Victoria Australia; ^27^ Department of Neuropediatrics The Children's Hospital at Westmead, Sydney Children's Hospital Network Sydney New South Wales Australia; ^28^ New South Wales Health Pathology Randwick Genomics Laboratory Sydney New South Wales Australia; ^29^ Department of Medical Genetics and Cytogenetics Limoges University Hospital Limoges France; ^30^ Univ Angers, INSERM, CNRS, MITOVASC, Equipe MitoLab, SFR ICAT Angers France; ^31^ Department of Genetics University Medical Center Utrecht Utrecht The Netherlands; ^32^ Department of Neurology Royal Children's Hospital Melbourne Victoria Australia; ^33^ Department of Medical Genetics University Hospital of Reims Reims France

## Abstract

**Objective:**

We aimed to characterize the phenotypic spectrum and functional consequences associated with variants in *HCN2*, encoding for the hyperpolarization‐activated cyclic nucleotide (HCN) gated channel 2.

**Methods:**

GeneMatcher facilitated the recruitment of 21 individuals with *HCN2* variants from 15 unrelated families, carrying *HCN2* variants. In vitro functional studies were performed by electrophysiology with *Xenopus laevis* oocytes and membrane trafficking was investigated in HEK cells by confocal imaging. Structural 3D‐analysis of the *HCN2* variants was performed.

**Results:**

The phenotypic spectrum included developmental delay/intellectual disability (DD/ID, 17/21), epilepsy (10/21), language disorders (16/21), movement disorders (12/21), and axial hypotonia (10/21). Thirteen pathogenic variants (12 new and 1 already described) were identified: 11 missense (8 monoallelic and 3 biallelic), 1 recurrent inframe deletion (monoallelic), and 1 frameshift (biallelic). Functional analysis of p.(Arg324His) variant showed a strong increase of HCN2 conductance, whereas p.(Ala363Val) and p.(Met374Leu) exhibited dominant negative effects. The p.(Leu377His), p.(Pro493Leu), and p.(Gly587Asp) variants rendered HCN2 electrophysiologically silent and impaired membrane trafficking. Structural 3D‐analysis revealed that, except for p.(Arg324His), all variants altered HCN2 stability.

**Interpretation:**

Our findings broadened the HCN2 disease clinical spectrum to include DD/ID with or without epilepsy. Functional analysis in cellular models reveal that pathogenic *HCN2* variants can cause either loss‐of‐function or gain‐of‐function, providing critical information for the development of targeted therapies for *HCN2*‐related disorders. ANN NEUROL 2025;98:573–589

Diseases resulting from dysfunction of ion channels, collectively known as channelopathies, often manifest as neurodevelopmental disorders associated with epilepsy.^1^, ^2^, ^3^ Among the ion channels involved in epilepsy, the hyperpolarization activated cyclic nucleotide (HCN) gated ion channels have been highlighted as important regulators of neuronal excitability in disease for more than 2 decades.^4^, ^5^, ^6^, ^7^ HCN channels are composed of 4 subunits forming a central ion‐conducting pore and assembled in homo‐ or heterotetramers.^4^, ^8^ HCN1 to 4 channels open upon hyperpolarization of the membrane to generate a non‐selective Na^+^/K^+^ currents, called I_h_ and I_f_ in neurons and cardiomyocytes, respectively.^4^ At resting potentials, this results in an inward current leading to membrane depolarization and exhibiting pacemaker activity in excitable cells. HCN channels are potentiated by intracellular cyclic monophosphate nucleotides cAMP and cGMP, through a positive shift of activation range.^4^, ^9^ Thereby HCN channels are involved in resting potential regulation, dendritic integration, and synaptic transmission.^4^

Of the 4 HCN genes (*HCN1–4*), *HCN1* and *HCN2* are the most abundant and widely expressed isoforms in the brain, whereas *HCN3* is predominant in the cerebellum, and *HCN4* is weakly expressed in the brain but highly abundant in the heart.^4^, ^10^ Several studies performed on transgenic mice have established a relationship between *HCN1* and *HCN2* and neuronal excitability and epileptogenesis, and being implicated in various neurophysiological processes, including sleep, learning, memory, and sensory functions.^11^, ^12^, ^13^, ^14^ *HCN1* variants are associated with a broader clinical spectrum, including developmental and epileptic encephalopathy (DEE) (DEE24; # 615871), epilepsy without intellectual disability (ID), and ID without seizures.^15^, ^16^, ^17^, ^18^, ^19^, ^20^

To date, 6 *HCN2* variants have been reported in a restricted number of individuals, with mild epilepsies including GGE and GEFS+, mostly without ID (Fig 1A).^21^, ^22^, ^23^, ^24^ Only 2 individuals with *HCN2* variants had ID: 1 of the 3 individuals from the family carrying the p.(Val246Met) variant, and 1 girl presenting ID and epilepsy, caused by a de novo p.(Gly460Asp) variant.^24^, ^25^ Functional studies have revealed that most *HCN2* variants, p.(Pro719‐Pro721del; previously reported as delPPP), p.(Ser632Trp), and p.(Val246Met) produce a gain‐of‐function (GoF) of the HCN2 channel, whereas 2 variants, p.(Glu515Lys) and p.(Gly460Asp), have been reported to cause loss‐of‐function (LoF).^21^, ^22^, ^24^, ^25^

**FIGURE 1:**
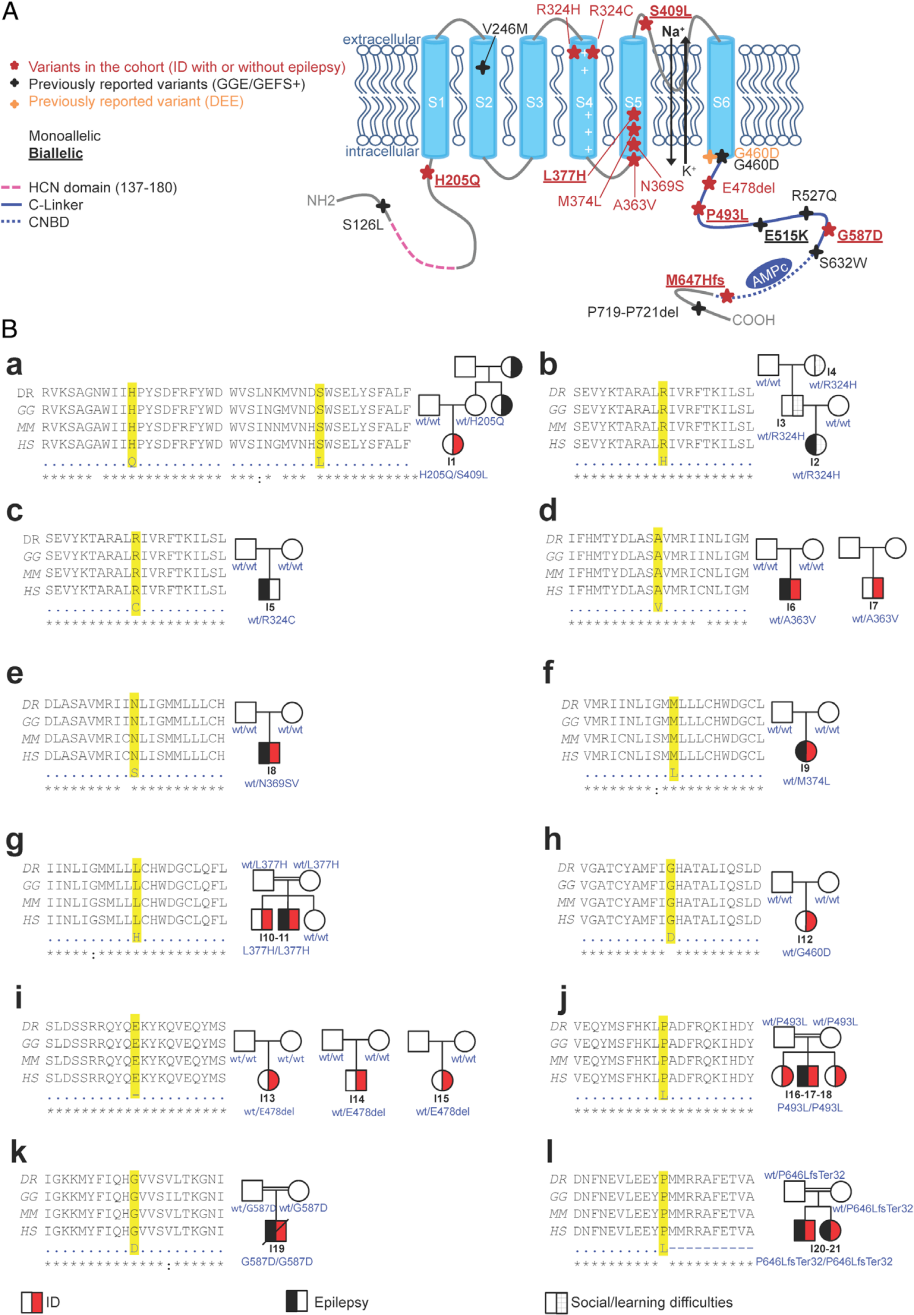
Pedigrees and *HCN2* pathogenic variants. (A) HCN2 channel and pathogenic variants. Stars indicate HCN2 variants identified in this work, and crosses indicate those previously reported. Biallelic variants are underlined whereas monoallelic variants are not. (B) Pedigrees of individuals I1 to 21 and multi‐species sequence alignment of the mutated HCN2 channels (a–i). Sequence alignment was performed using Clustal Omega (Uniprot entries: A0A8N7T6V3 [*Danio rerio*], A0A8V1AGR5 [*Gallus gallus*], O88703 [*Mus musculus*], Q9UL51 [*Homo sapiens*]). Below the sequence alignment, a key denotes: conserved amino acid (_*_), conservative replacement (:), and non‐conservative replacement (). When not mentioned, the individual's genotype is unknown. [Color figure can be viewed at www.annalsofneurology.org]

In this study, we report 21 individuals from 15 unrelated families, carrying mono‐ or biallelic *HCN2* variants. By characterizing the biophysical properties of 6 variants by 2‐electrode voltage‐clamp method (TEVC), Western blot analysis, membrane trafficking analysis, and 3D modeling, we demonstrate that *HCN2* variants are associated with a more severe and broader clinical spectrum of neurodevelopmental disorders than previously reported, and confirm its involvement in DEE.

## Subjects/Materials and Methods

### Ethics

This study was performed in accordance with ethical principles for medical research outlined in the Declaration of Helsinki and was approved by the ethics committee from the University Hospital of Angers (2022‐123). Written informed consents for genetic analyses were obtained from all individuals or their legal guardians. All animal procedures were performed in accordance with the European Community council directive 2010/63/EU for the care and use of laboratory animals, and approved by the French Ministry of Agriculture (authorization No. A49007002 and APAFIS N#19433‐2019022511329240v2). The NC3R's ARRIVE guidelines were followed in the conduct of all experiments using animals.

### Individual Recruitment and Clinical Phenotyping

Individuals harboring *HCN2* pathogenic or likely pathogenic variants were recruited through an international collaborative effort facilitated by GeneMatcher.^26^ All the individuals were addressed in the context of a neurodevelopmental disorders, including developmental delay (DD), ID, or epilepsy, and were evaluated by a neurologist, a neuropediatrician, or a geneticist with expertise in neurodevelopmental disorders. Individuals were enrolled from epilepsy and genetic centers located in Australia, Denmark, France, Germany, the Netherlands, Saudi Arabia, the United Kingdom, and the United States. Physicians recorded clinical, electroencephalogram (EEG), neuroimaging, and genetic data using a dedicated form. Epilepsy syndromes and seizure type were classified according to the International League Against Epilepsy (ILAE) guidelines.^27^ The detailed genotypic and phenotypic of these 21 individuals are available in the Supporting Information (Supplementary Tables S1–S3).

### Genetic Analysis

Genetic analyses were performed in a diagnostic or research laboratory in all individuals, and, when available, in their parents. *HCN2* variants were identified using either proband‐only or Trio‐exome or genome sequencing and confirmed by Sanger sequencing. Variants were classified according to American College of Medical Genetics and Genomics (ACMG) criteria (Supplementary Table S4).^28^ The consequences of *HCN2* variants were interpreted on isoform NM_001194.4.

### *HCN2* Mutagenesis and In Vitro Transcription

A synthetic wild‐type HCN2 cDNA (Genecust, Boynes, France) corresponding to the coding wild‐type sequence of *HCN2* (wt‐HCN2; NM_001194.4) was inserted between the *BamH*I and *Hind*III sites in pGEM‐HEJUEL, an expression vector suitable for *Xenopus laevis* oocyte expression. The *HCN2* variants were generated by site‐mutagenesis, and their sequences were verified by Sanger sequencing. The mRNAs were prepared from linearized cDNA templates, as previously described.^29^ The p.(Gly587Asp) variant was constructed and transcribed in the Reid laboratory (Florey, Australia) according to published methods.^27^

### Oocyte Preparation and mRNA Injection

Adult *Xenopus laevis* female subjects were used for oocytes collection, as previously described.^29^ In vitro functional studies were conducted using the TEVC technique in *Xenopus* oocyte. Non‐injected oocytes were used as the negative control. Oocytes were injected with 21 ng of mRNA (wt‐HCN2 in control experiments or biallelic variants). To mimic heterozygosity, co‐expression experiments with equal amounts (10.5 ng) of wt‐*HCN2* and *HCN2* variant mRNAs (eg, wt/p.(Arg324His)) were performed.

### Two‐Electrode Voltage Clamp Recording

Details of the procedures and analysis used for TEVC recordings are given in the Supporting Information. From a holding potential, V_h_ = −30 mV, currents were elicited with a 2‐step protocol, comprising: (i) an activation step in 10 mV increments from −140 to −30 mV for 8 seconds, and (ii) a return to −130 mV for 3 seconds. Currents were analyzed using Clampfit version 10.7 (Molecular Devices).

### Western Blot Analysis

Details of Western blotting procedures are available in the Supporting Information. Thirty μg of protein from pooled oocytes, control, and experimental (equivalent to protein from 80%–100% of protein from one oocyte) were separated by electrophoresis on 8% poly‐acrylamide SDS‐PAGE gels. Proteins were transferred to nitrocellulose and the blots were blocked with a milk‐containing solution before incubation with 1:500 rabbit anti‐HCN2 APC‐030 (Alomone Labs; Cat# APC‐030, RRID: AB2313726). Beta‐actin (Thermo Fisher Scientific; Cat# PA1‐183, RRID: AB_2539914) was used as a loading control. Quantitative analysis, for example, densitometry, on the pooled samples was precluded by expression variability in individual oocytes.

### Transient Expression in HEK293 Cells and Confocal Fluorescent Microscopy

Details of procedure are available in the Supporting Information. Three independent transfections were performed involving 3 wells/transfection/mutant. HEK293T cells were transfected with N‐terminal EGFP tagged constructs encoding human wt‐HCN2, p.(Leu377His), p.(Pro493Leu), and p.(Gly587Asp). As the negative control, a truncated HCN2_ΔC‐X_ (Thr553Ter), a trafficking‐deficient construct, was used.^30^ The membrane was labeled with CellMask Orange Plasma Membrane. All images were acquired using Zeiss LSM900 microscope (Florey Neuroscience Microscopy Facility). Over‐view images were acquired using 20×/0.8 NA objective with 3 repeats per group. Higher resolution and images for analysis of relative sub‐cellular expression pattern, membrane versus cytosol, was done using high‐resolution 3D images acquired with Airyscan technique and C Plan‐Apochromatic 63×/1.4 NA oil immersion objective (n = 19–24 cells). Images were captured as 16 Bits, XY = 0.035 μm pixel size and Z step size = 0.13 μm. For quantitative image analysis, all acquisition parameters were matched across all samples. The imaging parameters were cell mask orange (CMO) for membrane fluorescence signal (Em/Ex 556/572); EGFP‐tagged constructs (Em/Ex 488/509) and DAPI nuclear stain (Em/Ex 353/465) were detected with a GaAsP‐PMT detector type, and wavelength detection ranges CMO:540–700; EGFP:490–545, and DAPI:400–487, respectively. Relative‐quantification of GFP expression was done using Image‐J software.^31^ Briefly, 2 regions of interest (ROIs) for each cell, membrane, and cytosol, were delineated. The membrane perimeter was defined using CMO and the remaining area assigned as cell cytosol. The mean GFP‐signal intensity was then obtained for each ROI.

### Molecular Modelling and Structural Analysis

The human HCN1 structures in depolarized (PDB 5U6P) and hyperpolarized (PDB 6UQF) states were used to build HCN2‐wt 3D‐models with MODELLER version 9.17 (see Supporting Information).^32^, ^33^ These 3D models were used as templates for modeling all variants. PyMOL (Molecular Graphics System, version 1.8; Schrödinger, LLC) was used to inspect all 3D models and to determine solvent accessible surface area (ASA) and the relative solvent accessibility (RSA). DDMut (https://biosig.lab.uq.edu.au/ddmut/) and DynaMut (https://biosig.lab.uq.edu.au/dynamut/) servers were used to compute the Gibbs free energy in depolarized and hyperpolarized states (ΔΔG_d_ and ΔΔG_h_).^34^, ^35^ ΔΔG<0 indicates destabilizing effects, whereas ΔΔG>0 predicts stabilizing effects.

### Graphs and Statistical Analysis

All graphs and statistical analyses were performed using GraphPad Prism version 7.02 software (La Jolla, CA, USA). Data are mean ± standard error of the mean (SEM). The normality of sample distribution was assessed with the Shapiro–wilk test and, as appropriate, differences between groups were analyzed with parametric or nonparametric tests, with significance at *p* < 0.05.

## Results

### Identification of Monoallelic and Biallelic Variants in HCN2

We identified 13 different variants in the coding sequence of *HCN2*, 12 were novel, including 11 missense variants, 1 recurrent inframe deletion (I13‐15), and 1 frameshift variant. The distribution of the HCN2 variants along the protein sequence is shown in Figure 1A. Among these variants, 7 were monoallelic, including 5 de novo, 1 was not maternally inherited and 1 was inherited over 3 generations. The remaining 6 variants were biallelic, including 4 homozygous and 2 compound heterozygous. Noteworthy, the compound heterozygous variants resulted from the unusual combination of one de novo variant (p.(Ser409Leu)) and one variant in trans (p.(His205Gln)), inherited from the asymptomatic mother (Fig 1B). The p.(Glu478del) variant was found recurrently in 3 unrelated individuals, and 2 variants, namely p.(Arg324Cys) and p.(Arg324His), involved the same amino acid. The p.(Gly460Asp) variant was previously described as a DEE causing variant.^25^ All 13 variants have in silico prediction scores in favor of a deleterious effect (see Supplementary Table S4). They all affect an amino acid that is highly conserved across all HCN subtypes in humans and other species (see Fig 1B) and are absent or extremely rare in the gnomAD database. Moreover, they all are clustered in the S4 to S5 segments, the C‐linker, and the cyclic nucleotide binding domain (CNBD) with no obvious correlation with the mode of inheritance.

### Clinical Features: Neurodevelopmental Phenotypes and Movement Disorders

The clinical data of the 21 individuals (11 female subjects and 10 male subjects) reported here, whose ages ranged from 2 to 61 years (median age = 8 years and 2 months), are summarized in the Table 1 and their complete descriptions are available in the Supporting Information (see Supplementary Tables S1–S3). The DD/ID was present in 17 of 21 individuals, and was of variable severity: mild (3/17, 18%), moderate (3/17, 18%), and severe to profound (11/17, 65%). Developmental regression was reported in 4 individuals and was temporally related to epilepsy in 1 individual.

**TABLE 1:** Main Clinical Characteristics of 21 Individuals With *HCN2* Variants

Ind.	Age* (Sex)	HCN2 Variant	DD/ID Severity	Independent Walking	Tone	Movement Disorder	Epilepsy (age)	Language
I1	2 y (F)	p.(His205Gln) // p.(Ser409Leu)	+++	No	Axial hypotonia	Dystonia, Sandifer syndrome	No	Singles words
I2	9 y (F)	p.(Arg324His)	BL	Yes	Axial hypotonia	No	Yes (3 y)	Appropriate then regression
I3	/ (M)	p.(Arg324His)	BL	Yes	/	/	No	/
I4	/ (F)	p.(Arg324His)	BL	Yes	/	/	No	/
I5	13 y (M)	p.(Arg324Cys)	No	Yes	Normal	No	Yes (5 y)	Normal
I6	44 y (M)	p.(Ala363Val)	++	Yes (limited)	Pyramidal signs	Yes (not detailed)	Yes (9 mo)	Delayed
I7	61 y (F)	p.(Ala363Val)	+	/	Pyramidal signs	Static cerebellar signs	No	Delayed
I8	42 y (M)	p.(Asn369Ser)	+	/	Normal	No	Yes (6 y)	Needed speech therapy
I9	8 y (F)	p.(Met374Leu)	++	Yes (18 m)	Normal	No	Yes (11 mo)	Delayed
I10	8 y (F)	p.(Leu377His)	+++	No	Pyramidal signs	Hand stereotypies	No	Nonverbal
I11	5 y (M)	p.(Leu377His)	+++	No	Axial hypotonia	No	Yes (2.5 mo)	Nonverbal
I12	2 y(F)	p.(Gly460Asp)	++	No	Axial hypotonia	No	Yes (6 mo)	Single words
I13	11 y (F)	p.(Glu478del)	+	No	Axial hypotonia	Dystonia, tremor	No	Few words, Makaton, able to read
I14	4 y (M)	p.(Glu478del)	+++	No	Axial hypotonia	No	No	No words, vocalizes
I15	5 y(F)	p.(Glu478del)	+++	No	Spasticity	Dystonia, stereotypies	No	No words, vocalizes
I16	14 y (F)	p.(Pro493Leu)	+++	No	Pyramidal signs	Dystonia, hand stereotypies	No	Nonverbal
I17	11 y	p.(Pro493Leu)	+++	No	Pyramidal signs	Dystonia	Yes (3 y)	Nonverbal
I18	8 y (F)	p.(Pro493Leu)	+++	Yes (7 y)	Axial hypotonia	Dystonia, hand stereotypies, tremor	No	Nonverbal
I19	6 y (M)	p.(Gly587Asp)	++++	No	Axial hypotonia	Stereotypies	Yes (4 mo)	Nonverbal (4 y)
I20	10 y (M)	p.(Met647His fsTer31)	+++	No	Axial hypotonia	No	Infancy	Nonverbal
I21	8 y (F)	p.(Met647His fsTer31)	+++	Yes (4 y)	Axial hypotonia	No	Yes (1 mo)	Single words

a = age at last review; BL = borderline; DD/ID = developmental delay/ intellectual deficiency score; F = female; Ind. = individual; M = male; m = months; Mat. Inh. = maternally inherited; ND = not determined; No = absence of feature; Pat. Inh. = paternally inherited; y: years; / = unknown; // = trans‐variants; + = mild; ++ = moderate; +++ = severe; ++++ = severe to profound.

Twelve individuals (57%) had seizures, 2 with febrile seizures (FS) only and 10 (48%) with epilepsy. The median age of epilepsy onset was 10 months (range = 1 month to 6 years). A range of seizure types and EEG patterns were observed. One individual had infantile epileptic spasms syndrome, 1 had epileptic encephalopathy with spike‐wave activation in sleep (EE‐SWAS), and the remaining patients had unclassified epilepsies. No consistent treatment response was identified. Seizures were ongoing at last review in 3 of 10 individuals.

**FIGURE 2:**
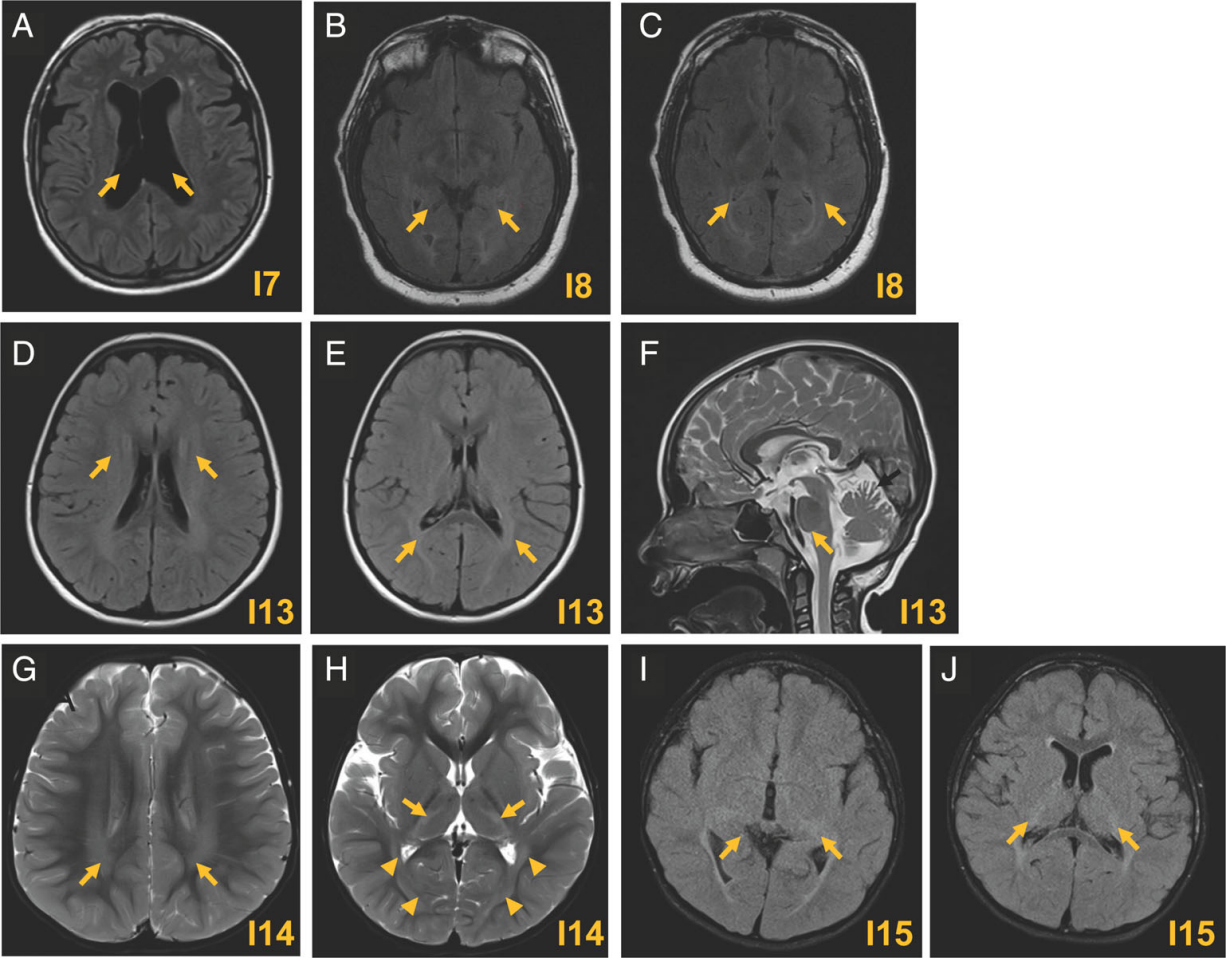
Cerebral MRI from individuals carrying the p.(Ala363Val), the p.(Asn369Ser), and the p.(Glu478del) variants. (A) Axial FLAIR MRI image from individual I7 show atrophy and an increase of ventricular spaces (arrows). Individual I7 carries the p.(Ala363Val) variant. (B, C) Axial brain MRI images show mild T2 signal changes in the periventricular white matter posteriorly in individual I8, carrying the p.(Asn369Ser) variant. (D–H) Axial MRI scans of individuals carrying the p.(Glu478del). (D–F) Axial FLAIR MRI images of individual I13 show periventricular white matter abnormalities. (F) Sagittal T2‐weighted image shows superior vermis atrophy (arrow C). (G, H) Axial T2‐weighted image through the centrum semiovale of individual I14 demonstrates a hyperintense signal involving the periventricular white matter with posterior predominance (arrows D). T2‐weighted signal hyperintensity is also present in the ventrolateral thalami (E arrows), as well as the optic radiations and subcortical occipital white matter (E arrow heads). (J, K) Axial T2‐weight images demonstrate posterior mild diffuse periventricular hyperintensity signal abnormality that extended into the posterior capsule into the thalami bilaterally in individual I15. FLAIR = fluid‐attenuated inversion recovery; MRI = magnetic resonance imaging. [Color figure can be viewed at www.annalsofneurology.org]

Tone abnormalities (hypotonia or pyramidal signs) were present in 16 individuals (76%; see the Table 1). Ten individuals had movement disorders, including dystonia (n = 6), stereotypies (n = 5), static cerebellar signs (n = 1), and tremor (n = 2). Nystagmus was present in 8 individuals (38%). Thirteen individuals (I1, I9–I11, and I20–21) exhibited a normal brain MRI, whereas abnormal brain MRIs were observed for 9 individuals (I2, I5–8, I13–15, and I19; Fig 2 and see Supplementary Tables S1–S3). Brain MRI showed white matter T2 hyperintensities in 5 individuals (I2, I7, and I13–15), including the 3 individuals carrying the p.(Glu478del) variant. Follow‐up MRIs will be necessary to determine the evolution of these anomalies over time.

### In Vitro Functional Analysis of Monoallelic HCN2 Variants

**FIGURE 3:**
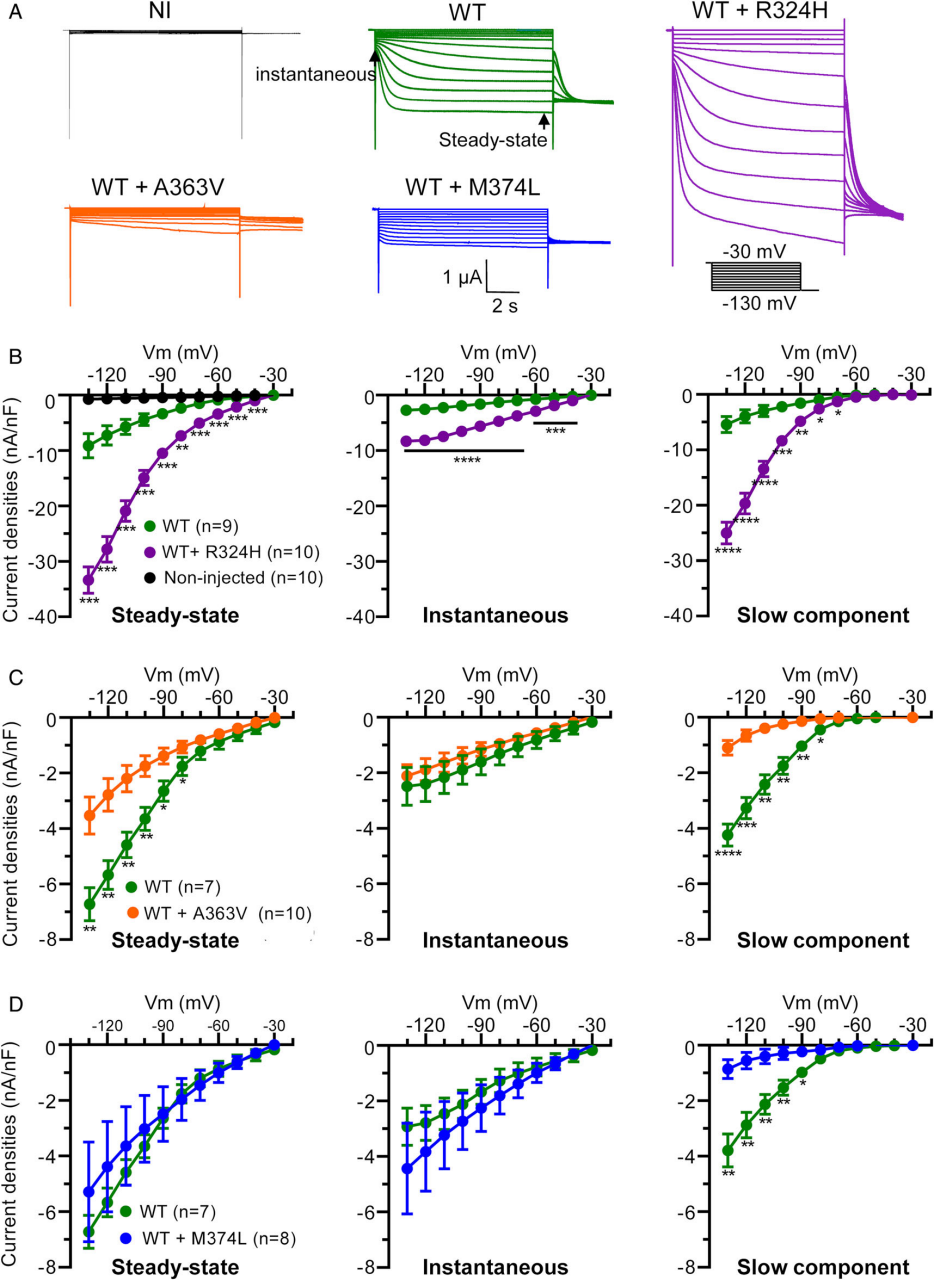
Electrophysiological characterization of the monoallelic variants, p.(Arg324His), p.(Ala363Val), and p.(Met374Leu). (A) Examples of superimposed current traces, developed in response to hyperpolarized stimulations. From left to right, are examples of currents elicited by uninjected oocytes (NI), wt‐HCN2 channel (WT), wt/p.(Arg324His) (WT + R324H), wt/p.(Ala363Val) (WT + A363V), and wt/p.(Met374Leu) (WT + M374L) channels, respectively. (B–D) From left to right, the I/V curves were generated using current densities measured at the steady‐state and instantaneous components. Current densities for the slow component were then calculated. For each variant, significance of difference with wt‐HCN2 was analyzed with a 2‐way ANOVA test (corrected with Geisser–Greenhouse method), followed by a comparison test (2‐stage step‐up method of Benjamini, Kriger, and Yekutieli). Data are shown as mean ± SEM. For clarity, no information indicates no significance. *, *p <* 0.05; **, *p <* 0.01; ***, *p <* 0,001; ****, *p <* 0.0001. ANOVA = analysis of variance; WT = wild type. [Color figure can be viewed at www.annalsofneurology.org]

The TEVC recordings were performed on *Xenopus* oocytes, injected with mixtures of p.(Arg324His) or p.(Ala363Val) or p.(Met374Leu), and wt‐HCN2 mRNAs to mimic heterozygosity. For non‐injected oocytes (negative control), only rectangular currents were induced by voltage steps, attributable to endogenous leak currents (Fig 3A, B). Currents elicited by wt‐HCN2‐expressing oocytes 2 components: an instantaneous, and a slowly activating phase reached a steady‐state (see Fig 3A), as already described.^36^ The current families generated by the wt/p.(Arg324His) variant appeared similar in shape to that of wt‐HCN2, but with apparent larger amplitudes (see Fig 3A). In contrast, the current families of wt/p.(Ala363Val) and wt/p/(Met374Leu) mixtures exhibited smaller amplitudes and different shapes (see Fig 3A). Concerning wt/p.(Arg324His), the current densities of steady‐state, instantaneous, and slow components were significantly higher (3 to 5‐fold) than those of wt‐HCN2 from −130 to −70 mV (see Fig 3B). In contrast for wt/p.(Ala363Val) and wt/p.(Met374Leu) combinations, we observed profound modifications of the current shape in comparison with wt‐HCN2 (see Fig 3A). The instantaneous components elicited by voltage steps for these 2 variants were similar to those of wt‐HCN2 (see Fig 2C, D). The wt/p.(Ala363Val) variant displayed significantly lower current densities for both steady‐state and slow components than wt‐HCN2 from −130 to −80 mV (Fig 3C). For the p.(Met374Leu) variant, we observed lower current densities of the slow component only (Fig 3D). Then, wt/p.(Ala363Val) and wt/p.(Met374Leu) variants induced a 5 and 3‐fold decrease of the slow component of HCN2 currents, respectively.

**FIGURE 4:**
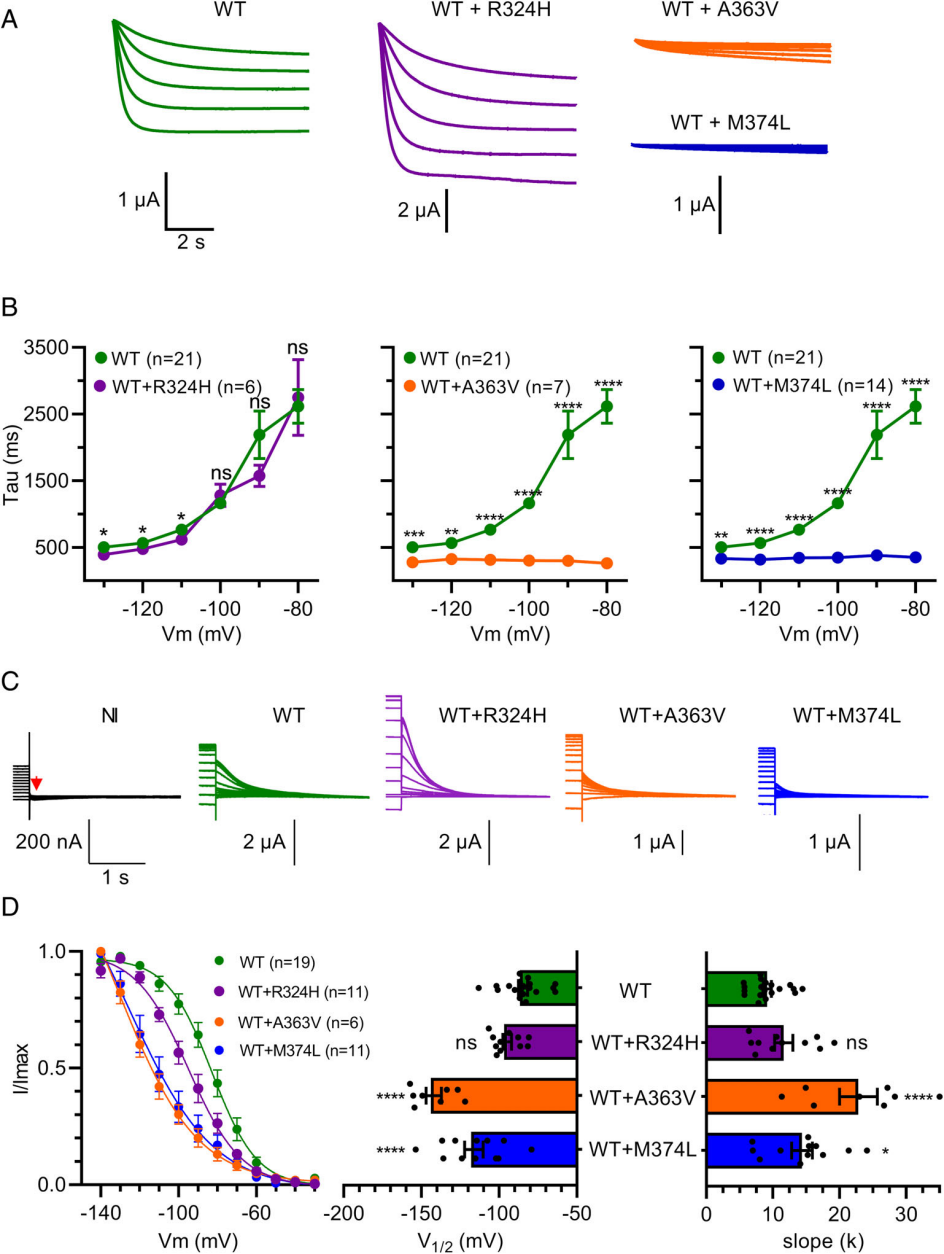
Activation kinetics and tail current analysis for the monoallelic variants, p.(Arg324His), p.(Ala363Val), and p.(Met374Leu). (A) Examples of traces showing the slow component of currents elicited by wt‐HCN2 channel (WT), wt/p.(Arg324His) (WT + R324H), wt/p.(Ala363Val) (WT + A363V), and wt/p.(Met374Leu) (WT + M374L) channels, respectively. (B) Activating current kinetics analysis. Current traces were well‐fitted with a single exponential equation. Tau (ms) was plotted versus Vm (mV). For each variant, significance of difference with wt‐HCN2 was analyzed with a 2‐way ANOVA test (corrected with Geisser–Greenhouse method), followed by a comparison test (2‐stage step‐up method of Benjamini, Kriger, and Yekutieli). (C) Representative tail current traces for wt‐HCN2 channel (WT), wt/p.(Arg324His) (WT + R324H), wt/p.(Ala363Val) (WT + A363V), and wt/p.(Met374Leu) (WT + M374L) channels, respectively. (D) The left panel shows the normalized current amplitudes (I/Imax) versus Vm were fitted with the Boltzmann equation (see Supporting Information), allowing the determination of the half‐activation potentials (V_1/2_) and the slope factor k. The right panel shows the scatter plots of the comparison of the V_1/2_ (left) and slope factor (right) of the wt‐HCN2, wt/p.(Ala363Val), wt/p.(Met374Leu), and wt/p.(Arg324His) mutant channels. A 1‐way ANOVA test (F = 53,15, *p <* 0.0001), followed by a Dunnett test, was performed and showed a significant difference between mutants and wt‐HCN2, except between the p.(Arg324His) and wt‐ HCN2 (*p* = 0.48). Data are shown as mean ± SEM. ns, not significant; *, *p <* 0.05; **, *p <* 0.01; ***, *p <* 0,001; ****, *p <* 0.0001. ANOVA = analysis of variance; WT = wild type. [Color figure can be viewed at www.annalsofneurology.org]

The slow component of wt‐HCN2 and wt/p.(Arg324His) current traces was the best fitted with a double exponential function, whereas those of both wt/p.(Ala363Val) and wt/p.(Met374Leu) variants were best fitted with a single exponential function, which was therefore used to analyze all traces (Fig 4A). The current traces of p.(Arg324His) variant exhibited slightly faster activation kinetics only from −130 to −110 mV (*p <* 0.05, Fig 4B). For both wt/p.(Ala363Val) and wt/p.(Met374Leu) variants, a significant increase in the rate of activation from −130 to −80 mV was observed in comparison to wt‐HCN2, which was all the more important as Vm was positive (see Fig 4B).

For the wt/p.(Arg324His) channel, the V_1/2_ and k‐values, determined from the instantaneous tail currents were not significantly different from those of wt‐HCN2 channel (Fig 4D). In contrast, for both the wt/p.(Ala363Val) and wt/p.(Met374Leu) channels, V_1/2_ of activation was shifted to the left by 60.8 and 43.0 mV, respectively (*p <* 0.0001; see Fig 4D). This hyperpolarized shift was significantly larger for the p.(Ala363Val) variant than for the p.(Met374Leu) variant (*p <* 0.0001; see Fig 4D, middle graph). For these variants, the slopes were significantly shallower than that of wt‐HCN2 (*p <* 0.0001; see Fig 4D).

Qualitatively, the Western blots did not show any obvious significant protein reduction for either variant (Supplementary Fig S1). Thus, these in vitro functional data are consistent with the p.(Arg324His) variant resulting in GoF, whereas the p.(Ala363Val) and the p.(Met374Leu) variants induce a partial LoF.

Interestingly, the co‐expression of the p.(Met374Leu) HCN2 variant with wt‐HCN1 induced a significant positive shift in the voltage‐dependency of tail currents, and produced cationic leakage, similar to what has been observed with the p.(Met305Leu) HCN1 variant, which alters the structure of HCN1 at the same location (Supplementary Figs S6 and S7).^37^, ^38^

### In Vitro Functional and Trafficking Analysis of Biallelic HCN2 Variants

**FIGURE 5:**
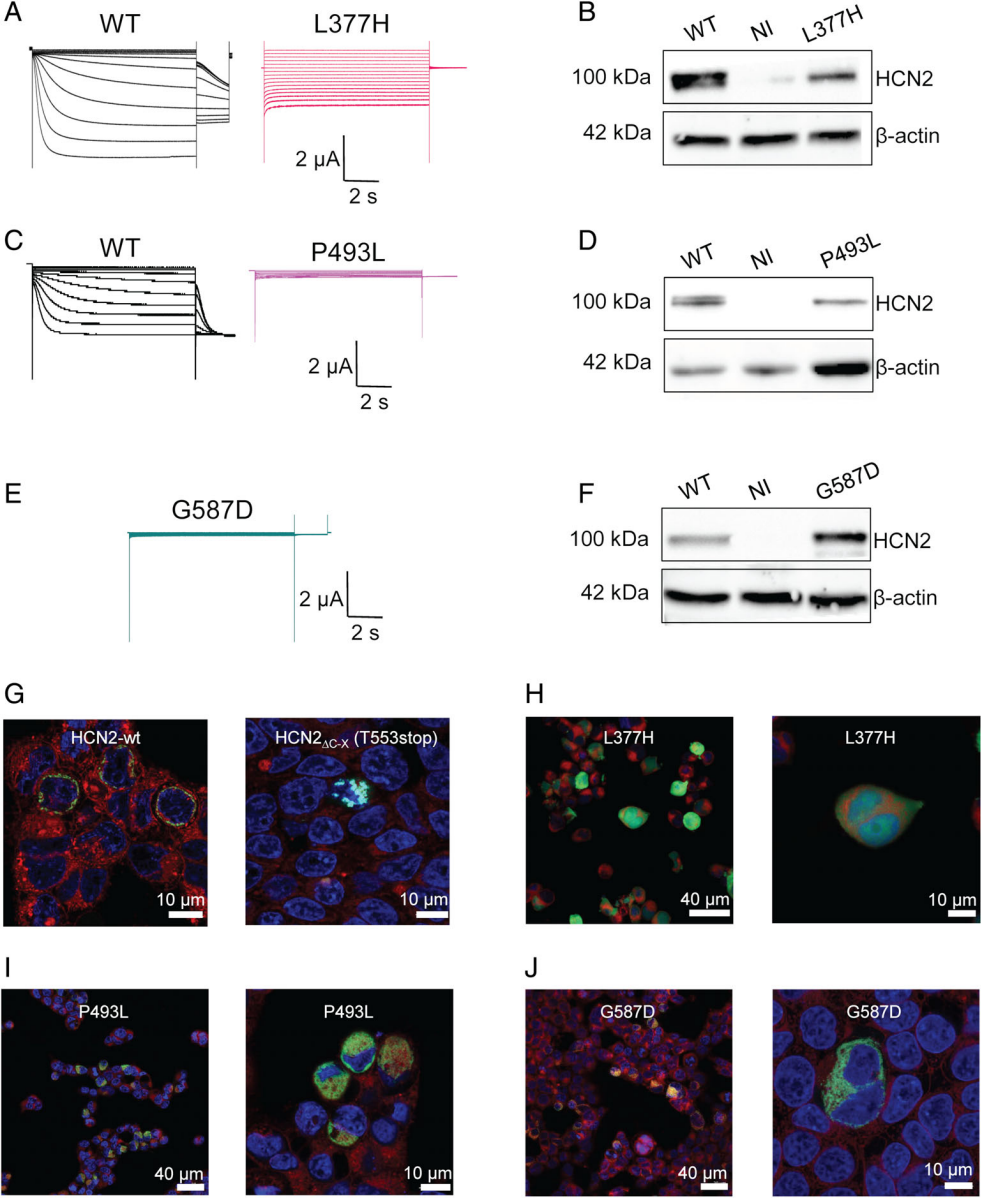
Characterization of the p.(Leu377His), the p.(Pro493Leu), and the p.(Gly587Asp) HCN2 variants. (A, C, and E) Representative TEVC recordings from oocytes expressing HCN2 wild‐type and 2 homozygous variants of HCN2, p.(Leu377His), p.(Pro493Leu), and p.(Gly587Asp). The data show current traces for voltage steps from −130 to −30 mV from a holding potential of −30 mV for 10 seconds. (B, D, and F) Western blot of *Xenopus laevis* oocyte proteins. The panels show immunostaining with anti‐HCN2 and anti‐β‐Actin antibodies. WT: Oocytes injected with wt‐HCN2 mRNA, NI: Non‐injected oocytes; L377H, P493L and G587D: Oocytes injected with the p.(Leu377His), the p.(Pro493Leu), and the p.(Gly587Asp) variants mRNAs, respectively. (G, H, J) Study of membrane trafficking of the p.(Leu377His), the p.(Pro493Leu), and the p.(Gly587Asp) variants in HEK293 cells. The expression of EGFP‐tagged the p.(Leu377His) or the p.(Pro493Leu) or the p.(Gly587Asp) HCN2 variants was evaluated by confocal microscopy. The cells were stained with the CellMask orange membrane stain and DAPi nuclear stain. EGFP‐tagged wt‐HCN2 (G, left panel) shows a strong EGFP signal at the plasma membrane, while the trafficking‐defected HCN2Δ_C‐X_ (Thr553Ter) shows cytosolic staining (G, right panel), as seen for the p.(Leu377His), the p.(Pro493Leu), and the p.(Gly587Asp) variants (H, I, J). Examples of images obtained from magnification of 63× are shown (G and H–J, left panels). Examples of overview with magnification from 20× are also shown (H–J, left panels). TEVC = 2‐electrode voltage‐clamp method; WT = wild type. [Color figure can be viewed at www.annalsofneurology.org]

The biallelic variants p.(Leu377His) (n = 8), p.(Pro493Leu) (n = 6), and p.(Gly587Asp) (n = 4) did not produce any measurable currents in *Xenopus* oocytes (Fig 5A, C, E). Nevertheless, Western blotting showed a strong immunoreactivity for bands for all 3 variants with apparent molecular weight close to 100 kDa (Fig 5B, D, F). Two bands immune‐reacted in wt‐HCN2, likely reflecting both glycosylated and non‐glycosylated forms as previously reported.^39^ However, p.(Leu377His) and p.(Pro493Leu) showed only one band corresponding to the non‐glycosylated form, whereas the p.(Gly587Asp) variant behaved as wt‐HCN2. As N‐glycosylation is required for HCN2 trafficking in the plasma membrane, we hypothesized that the LoF of *HCN2* variants, p.(Leu377His) and p.(Pro493Leu), could be the consequence of retention of the protein in the cytosol. We then expressed p.(Leu377His), p.(Pro493Leu), and p.(Gly587Asp) HCN2 variants, wt‐HCN2, and the negative control truncated HCN2_ΔC‐X_ (T553stop), all tagged with EGFP, in HEK293 cells and imaged their trafficking to the plasma membrane by confocal microscopy (Fig 5G–J). Although EGFP‐tagged HCN2‐wt showed clear staining of the plasma membrane (Fig 4G), no obvious membrane expression was observed for p.(Leu377His), p.(Pro493Leu), and p.(Gly587Asp) HCN2 variants (Fig 5G–J). Analysis of the relative expression in plasma membrane versus cytosol (Supplementary Fig S2), is consistent with all 3 biallelic variants having a significant trafficking deficiency at physiological temperatures.

Contrary to what was observed with the p.(Met374Leu) HCN2 variant, the p.(Gly587Asp) HCN2 variant had no obvious effects on wt‐HCN1 currents in *Xenopus* oocytes (see Supplementary Figs S6 and S7).

### Structural Impacts of HCN2 Variants

**FIGURE 6:**
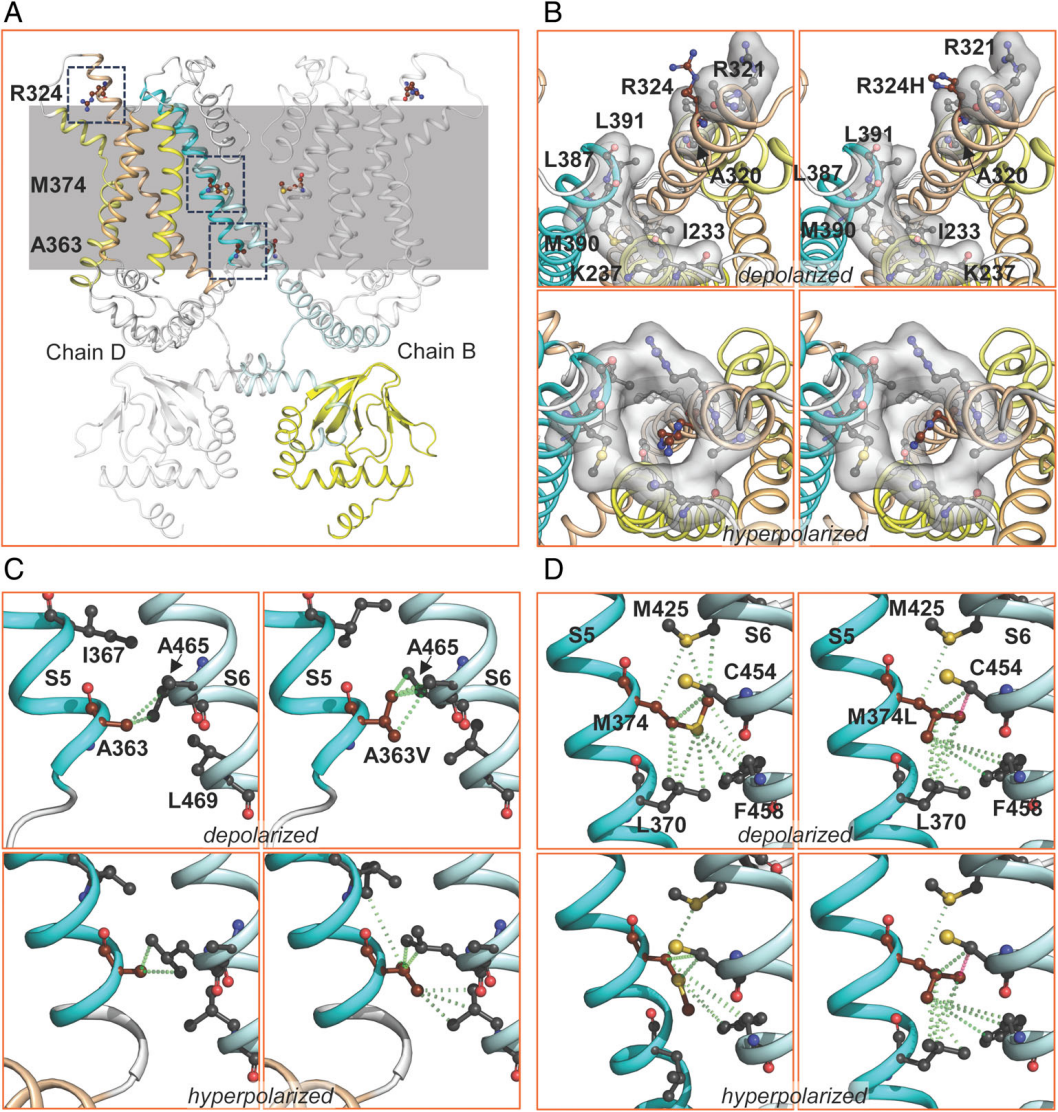
Structural analysis of the monoallelic pathogenic HCN2 variants, p.(Arg324His), p.(Ala363Val), and p.(M374Leu). (A) Mutation mapping onto 3D homology model of HCN2. Profile view of HCN2 3D structure in depolarized state, showing R324, A363, and M374 positions. For clarity, the chains A and C were hidden. The approximate position of HCN2 within the membrane is visualized with the gray rectangle. The regions delimitated by the dashed box were enlarged to illustrate the closest contacts of R324, A363, and M374 in chain B in the depolarized and hyperpolarized states (B, C, D). (B) Top views of R324 (in chocolate). The side chain of residue at 324 is toward the solvent in the depolarized state of HCN2, but, after opening, it remains trapped in a hydrophobic pocket formed by I233, K237, A320, R321, F328, L387, M390, and L391. The side chain of His fits well in this hydrophobic pocket. (C and D) Profile views of S5 to S6 interfaces showing the residues A363 and M374 and their hydrophobic interactions with their closest neighbor contacts. The hydrophobic interactions (in green dashed lines) are stronger in the p.(Ala363Val) and the p.(M374Leu) variants. In the p.(M374Leu) variant, a clash is observed between L374 and C454 (D). [Color figure can be viewed at www.annalsofneurology.org]

The 3D models of wt‐HCN2 and all HCN2 variants were examined in both depolarized and hyperpolarized states to decipher the structural impact of each mutation (Supplementary Figs S2–S5). The effects of each missense mutation on HCN2 structure stability were assessed by analyzing the ΔΔG_d/h_ values (Supplementary Table S5). The compound heterozygote (p.(His205Gln)/p.(Ser409Leu)) and biallelic GoF variants, (p.(Pro493Leu), and p.(Gly587Asp) exhibit ΔΔG_d/h_ < −2.7 kcal/mol, indicating high destabilizing effects on the depolarized state of HCN2. The p.(His205Gln)/p.(Ser409Leu) and p.(Gly587Asp) variants also show high destabilizing effects on the hyperpolarized state of HCN2 (ΔΔG_d/h_ < −1.9 kcal/mol). Whereas p.(Leu377His) and p.(Gly460Asp) variants have weak stabilizing effects on both depolarized and hyperpolarized states (0 > ΔΔG_d/h_ > −1 kcal/mol), in contrast, p.(Arg324His), p.(Arg324Cys), p.(Ala363Val), p.(Asn369Ser), and p.(Met374Leu) variants show positive ΔΔG values, indicating stabilizing effects on HCN2 structure. The GoF p.(Arg324His) variant is located in S4 and induced the replacement of the Arg324 side chain, that is freely exposed toward the solvent at the extracellular side in the depolarized state. Interestingly, during HCN2 opening, S4 rotates counterclockwise, burying the Arg324 side chain in a hydrophobic pocket (Fig 6A, B). The ASA values of Arg324 dropped from 139.27 Å^2^ (RSA = 64%) to 24.07 Å^2^ (RSA = 14.12%) between the depolarized state and the hyperpolarized state. In the p.(Arg324His) variant, the shorter side chain of His, at position 324, fitted better in this hydrophobic pocket in the hyperpolarized state (ASA = 14.12 Å^2^, RSA = 7%) and could be stabilized by π‐ interaction with Phe328 (see Fig 6B). This might explain the strong GoF in *Xenopus* oocytes. Similar assessment could be made with the p.(Arg324Cys) variant, because Cys is also more hydrophobic and smaller than Arg. The p.(Ala363Val) and p.(Met374Leu) variants alter hydrophobic interactions that stabilize the S5 to S6 conformation (Fig 6C, D). Replacement of Ala363 by Val, which is bulkier and more hydrophobic, could form a stronger hydrophobic interaction, stabilizing the hyperpolarized state (Fig 6C), in agreement with its faster activation kinetics in *Xenopus* oocyte. Similar statements could be made with the p.(Met374Leu) variant, for which stronger hydrophobic interactions between S5 and S6, might stabilize the hyperpolarized state (see Fig 6D). This is supported by the increase of ΔΔG values, and reorganization of the S4 to S5 segment during channel opening, as previously reported.^38^

**FIGURE 7:**
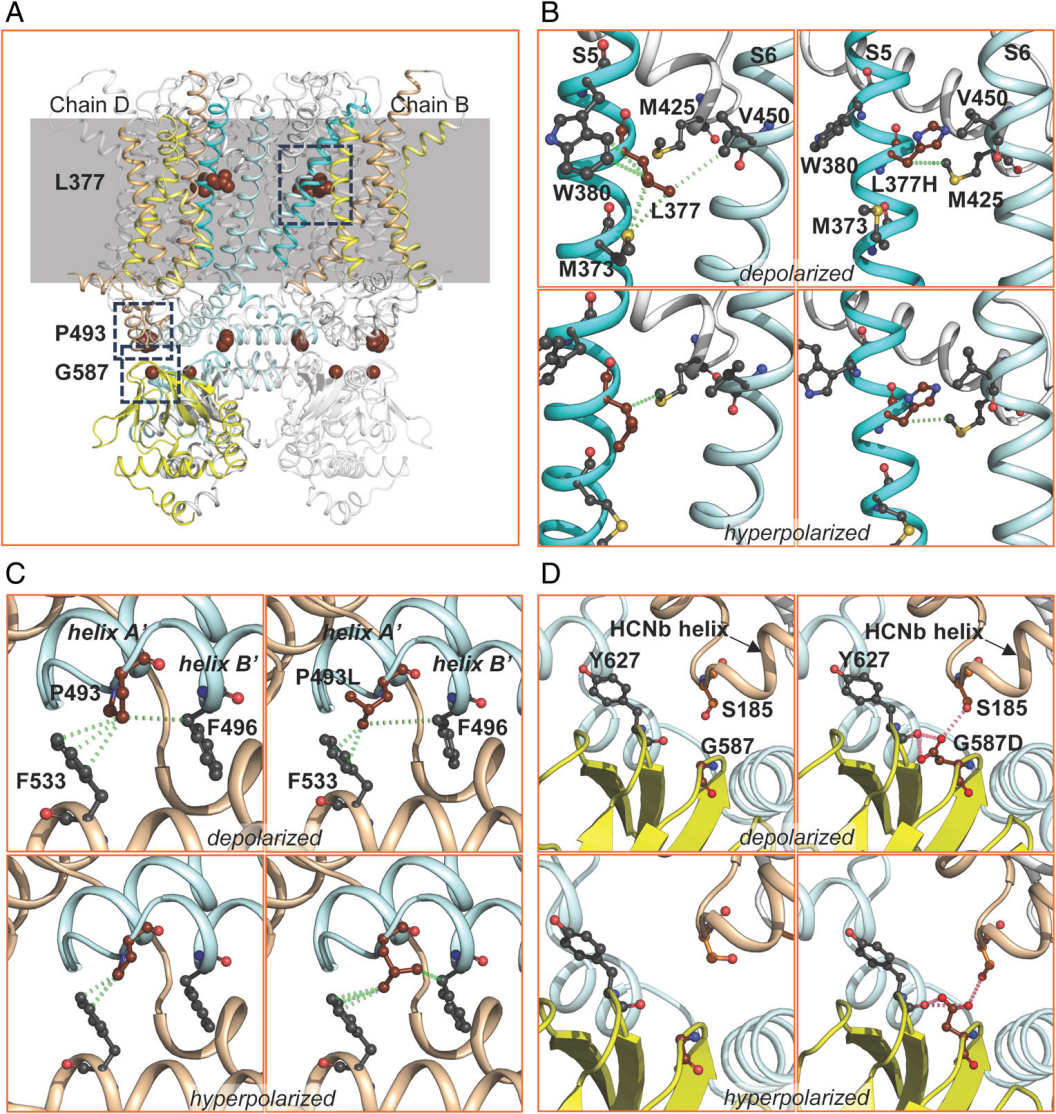
Structural analysis of the biallelic pathogenic variants, p.(Leu377His), p.(Pro493Leu) and p.(Gly587Asp). (A) Mutation mapping onto 3D homology model of HCN2. A profile view of HCN2 3D structure in depolarized stated shows L377, P493, and G587 positions. For clarity, only chains B and C were colored. The approximate position of HCN2 within the membrane is visualized with the gray rectangle. The regions delimitated by the dashed box were enlarged to illustrate the closest contacts of each amino acid residue in chain B (B, C, D). (B) Enlarged view of the S5 to S6 interface of wt‐HCN2 and p.(Leu377His) HCN2 variant in the depolarized and hyperpolarized states. In the depolarized state, the side chain of L377 is stabilized by hydrophobic interactions with M373 and V450 side chains at the interface between S5 (in cyan) and S6 (in light blue). After opening, in the hyperpolarized state, L377 forms only one hydrophobic interaction with M425, localized in the pore helix (in white). In p.(Leu377His), the hydrophobic interactions with M373 and V450 are lost and L377H interacts with M425 in both the depolarized and hyperpolarized states. (C) Enlarged view of the interface between chains B and C at the C‐termini of HCN2. P493 (chain B in the loop between helix A′ and B′) forms hydrophobic interactions with F533 (chain C) and F496 (helix B′, chain B) in the depolarized state. After opening, these hydrophobic interactions are weaker and concerns only F533. In the p.(Pro493Leu) variant, these hydrophobic interactions are partially lost, that might destabilize the bend between helices A′ and B′ and also the N‐terminal of helice B′. (D) Enlarged view of the interface between the CNBD of chains B and the N‐termini of chain C in the HCN2 structure. The p.(Gly587Asp) variant changes the curvature of the loop between 2 β‐sheets within the CNBD (in yellow). Several clashes occurred in the G587D and Y627 (chain B), and the S185 (chain C). The hydrophobic interactions are illustrated by green dashed lines and clashes are shown in pink. [Color figure can be viewed at www.annalsofneurology.org]

In the p.(Leu377His) variant, the replacement of Leu by His induced the loss of strong hydrophobic interactions at the interface between S5 and S6 (Fig 7A, B). However, we found that the His side chain exhibits a different orientation than Leu and could strongly interact with Asp381 through a salt bridge (see Fig 7B). These modifications support the strong destabilizing effects in the depolarized state of HCN2, and might account for the large leak currents associated with this substitution (see Fig 5A). In the C‐linker, and the p.(Pro493Leu) and p.(Glu587Asp) variants modify buried residues (see Fig 7A, C, D). The p.(Pro493Leu) variant destabilizes the kink between the A′ and B′ anti‐parallel helices (Fig 7C), whereas the p.(Gly587Asp) led to the formation of a salt bridge between the CNBD and the channel N‐terminus (Fig 7D). In summary, the p.(Leu377His), p.(Pro493Leu), and p.(Gly587Asp) variants have a major structural impact caused by polar and/or hydrophobic interactions changes, that might explain the destabilization of HCN2 structure and subsequent LoF.

### Phenotypic Differences Between Individuals With GoF and LoF Variants

In individuals carrying a GoF variant (n = 18, including the 3 individuals from family 2 and 15 individuals in the literature), seizures were present in 16 of 18 (89%, FS only in 8/16 and epilepsy in 8/16) with a median age of onset of 5 years, but intellectual disability was present in only 1 of 18 individuals (5.5%; see the Table 1 and Supplementary Tables S1 and S2). In individuals carrying the LoF variants (n = 12, individuals 6, 7, 9, 10, 11, 12, 16, 17, 18, and 19 from this cohort and 2 individuals from literature), epilepsy was present in 8 of 12 individuals (67%) with a median age of onset of 7.5 months. ID was present in 11 of 12 (92%) individuals (not stated in 1 individual), being of moderate severity in 2 individuals with monoallelic LoF, and severe to profound in 3 individuals with biallelic LoF (see Supplementary Table S2). All individuals (8/21) with LoF biallelic variants had severe DD/ID (Supplementary Table S3).

## Discussion

By describing 21 additional individuals with pathogenic *HCN2* variants, we expand the phenotype of *HCN2*‐related disorders, from mild epilepsies with normal cognition, to phenotypes, including ID with or without epilepsy, individuals with DEE, and movement disorders. We report 11 novel *HCN2* variants. In vitro functional characterization of 6 variants highlight that these variants can cause either GoF, by increasing HCN2 conductance or partial LoF, by altering HCN2 gating, or complete LoF, by impairing membrane trafficking. Our findings provide preliminary evidence of phenotype–genotype correlation: specifically, that LoF variants may be associated with more severe ID and, where epilepsy is present, earlier age of seizure onset.

Both the p.(Ala363Val) and the p.(Met374Leu) variants strongly alter HCN2 gating properties, when co‐expressed with wt‐HCN2 in *Xenopus* oocyte. Thereby, heterotetrameric ion channels composed of the p.(Ala363Val) and the p.(Met374Leu) variants with wt‐HCN2 exhibit lower conductance, faster kinetics, and open at more hyperpolarizing potential than wt‐HCN2. We attribute the changes to a partial LoF of HCN2. Thus, these observations highlight the dominant negative effect of the p.(Ala363Val) and the p.(Met374Leu) HCN2 variants. This assumption is reinforced by structural analysis, reinforcing the conclusion that Ala363 and Met374 are key residues in the S5 and S6 stability.

The p.(Met374Leu) variant is a homologous variant to the p.(Met305Leu) HCN1 variant, which has been associated with DEE.^37^, ^38^, ^40^ However, whereas the p.(Met374Leu) HCN2 variant shows similar current kinetics to the p.(Met305Leu) HCN1 variant, the instantaneous tail currents elicited by both variants shift in opposite direction.^37^ This could be explained by biophysical specificities of HCN1 versus HCN2. Interestingly, the p.(Met374Leu) HCN2 variant alters the function of wt‐HCN1 in *Xenopus* oocytes, like the p.(Met305Leu) HCN1 variant, by shifting the voltage‐dependency of tail currents toward positive potentials, and producing the cation leak at the depolarized potentials.^37^, ^38^ This suggests that both variants share similar pathogenic mechanisms, at least partially. Indeed, the individuals carrying the p.(Met374Leu) HCN2 or the p.(Met305Leu) HCN1 variants have epilepsy and neurodevelopmental disorders.^37^, ^38^, ^40^ As proposed for the p.(Met305Leu) HCN1 variant, blocking the aberrant p.(Met374Leu) HCN2/HCN1 leaking channels could be a therapeutical strategy to prevent epilepsy.^38^, ^40^

In contrast, the 3 biallelic variants, p.(Leu377His), p.(Pro493Leu), and p.(Gly587Asp) are electrophysiologically silent in *Xenopus* oocytes and induce strong destabilization of the HCN2 structure. Interestingly, these variants could not be detected at the plasma membrane, indicating that the p.(Leu377His), the p.(Pro493Leu), and the p.(Gly587Asp) variants impair the HCN2 membrane trafficking, likely due to HCN2 misfolding or loss of interaction with trafficking regulating proteins, such as TRIP8b.^30^ Alteration of the HCN2 membrane trafficking has also been reported for one monoallelic HCN2 variant (p.(Gly460Asp)).^25^ Contrary to the p.(Met374Leu) HCN2 variant, the p.(Gly587Asp) HCN2 variant shows no significant effect on wt‐HCN1. We postulate that the p.(Gly587Asp) HCN2 variant is most likely misfolded, and therefore cannot heterotetramerize with HCN1. Further experiments would be necessary to investigate the impact of the p.(Leu377His), and the p.(Pro493Leu) HCN2 variants on the HCN1 function, beyond the scope of the present study.

Our study brings novel evidence to clearly establish LoF as a mechanism of HCN2‐related disease, building on 2 previous case reports. As recently suggested by DiFrancesco, who reported that the p.(Gly460Asp) results in LoF, we believe that the pathogenicity of monoallelic LoF HCN2 variants is due to a dominant negative effect rather than haploinsufficiency, as evidenced by 3 observations.^25^ First, in our cohort (7 cases from 4 families) and the literature (1 case), the heterozygous parents of a child with a homozygous biallelic variant were all unaffected. Second, disease‐causing monoallelic truncating HCN2 variants have not been reported to date; similarly, truncating pathogenic variants have not been reported in *HCN1* or *HCN4*. Third, data from the gnomAD database includes 12 truncating *HCN2* variants in healthy individuals, and a pLI score of 0.17 indicating a low probability that *HCN2* could be intolerant to LoF.

We also provide data to advance understanding of GoF *HCN2* variants. The p.(Arg324His) variant induces an increase of HCN2 conductance and faster currents in *Xenopus* oocyte compared to wt‐HCN2. The structural analysis revealed an interesting feature of the residue at position 324. The side chain of Arg324 falls into a hydrophobic pocket during channel opening, which constitutes a more stabilizing environment for histidine and cysteine and likely favors the HCN2 open state. Thereby, the GoF caused by the p.(Arg324His) variant results from an important increase of HCN2 conductance as reported for the p.(Pro719‐Pro721del) variant, but also from faster activation kinetics, not seen for this latter, but for the p.(Val246Met) and the p.(Ser635Trp) monoallelic variants.^21^, ^24^

Similar to *HCN1*, our data and the literature allowed us to highlight limited genotype–phenotype correlation for HCN2‐related conditions (see the Table 1). Seizures were a common but not universal feature in those with either GoF or LoF variants, being present in 89% (16/18) and 67% (8/12) respectively. However, only 44% (8/18) individuals with GoF variants had epilepsy, the remaining individuals having FS only. Additionally, the age of epilepsy onset was earlier in those with LoF variants (median = 7.5 months) compared with GoF variants (median = 5 years). DD/ID was present more frequently and was more severe in those with LoF variants (present in 11/12 individuals [92%], range of moderate to severe) than with GoF variants (present in 1/18 individuals [6%], range of normal to mild). We note that features other than the *HCN2* variant likely contribute to disease severity: we saw intrafamilial variability in severity for family 2 with the monoallelic p.(Arg324His) variant and for families 7, 11, and 14 carrying the homozygous p.(Leu377His), p.(Pro493Leu), and p.(Met647HisfsTer31) variants, respectively. Some of these families (Fig 1Bb, Bg, Bj) include both individuals with and without epilepsy. Such variability for a variant has already been reported for other ion channel genes as for *SCN1A*.^41^ Additionally, we noted interindividual variability for the 2 individuals carrying the monoallelic p.(Ala363Val) variant in terms of ID severity and presence of epilepsy; and the 3 individuals carrying the inframe p.Glu478del variant, with variability in the severity of visual impairment and movement disorder.

Our data highlight that the related HCN2 phenotype ranges from febrile seizures or mild epilepsy without ID to severe forms of epilepsy with ID and ID without epilepsy.^16^ Thus, one characteristic shared by both isoforms *HCN1* and *HCN2* is the wide phenotypic spectrum caused by channel disruption. A relevant difference between *HCN1* and *HCN2* is the absence of reported biallelic pathogenic variants in *HCN1*, whereas biallelic inheritance is frequent in our HCN2 cohort. Another difference between *HCN1* and *HCN2* variants is the lack of movement disorders reported so far for individuals with the *HCN1* variants.

It is therefore interesting to compare the severity of the phenotype with the degree of impact of the variants on HCN2 function. Among the 12 individuals carrying the LoF variants, only one with the p.(Glu515Lys) variant has been described with normal neurodevelopment.^22^ However, the p.(Glu515Lys) variant has a less deleterious effect on the HCN2 currents in cell models, compared with the p.(Ala363Val), the p.(Met374Leu), the p.(Leu377His), the p.(Gly460Asp), the p.(Pro493Leu), and the p.(Gly587Asp) variants inducing near complete LoF.^22^, ^25^ The p.(Glu515Lys) variant is located in a negative ring, extending the permeation pathway and the modification of the charge‐ring amino acid affects gating without altering ion channel conductance.^22^ We speculated that the ID found in the other individuals is related to the altered HCN2 conductance. Further studies on neuronal cells and in vivo experiments will be needed to confirm this hypothesis.

Given that HCN2 coassembles with HCN1 in the brain, it is particularly important to also investigate whether variants of one affect the HCN1/HCN2 heterotetramer function.^4^, ^8^ Our preliminary data show that HCN1/p.(Met374Leu) HCN2 heterotetramers expressed in *Xenopus* oocytes have lost voltage dependence, as described for the HCN1/p.(Met305Leu) HCN1 variant.^37^, ^38^ In contrast, the p.(Gly587Asp) HCN2 variant appears to have no impact on the HCN1/HCN2 activity in *Xenopus* oocytes. Thus, each HCN2 variant might have distinct effects on HCN1/HCN2 heterotetrameric channel functions, and should be examined in further experiments. However, because HCN channels can heterotetramerize with different stochiometry, at different expression levels, in different neurons and neuronal sublocalization, information from in vitro experiments coassembling HCN2 variants with HCN1 is somewhat limited.

Although our cohort is the largest in terms of the number of individuals and *HCN2* variants reported to date, functional studies were not performed on all novel *HCN2* variants, and therefore cannot confidently determine whether they result in GoF or LoF. No other pathogenic variant in relation to the clinical phenotype were identified in the individuals reported here, but any genetic background effects or environmental impacts would not have been detected. Another limitation of our in vitro functional studies was the use of a single cell model, which specifically addresses the effects of variants on the HCN channels gating properties, but cannot include the overall effects on membrane excitability, nor on the brain in a whole animal model. As performed elsewhere, molecular dynamic simulation studies of the effect of these variants on the HCN2 channel 3D structure would provide additional information on the alteration of the gating mechanisms.^16^, ^37^ At a neuronal circuit and whole brain level, the implications of the HCN2 channel dysfunction are yet to be elucidated. The use of stem cell and organoid models, and generation of transgenic mice, will advance understanding of disease mechanisms, and provide models upon which to develop targeted therapies, as done recently for *HCN1*.^42^ More specifically, allele specific oligonucleotide antigens could be a promising treatment for monoallelic variants, as our data exclude the pathogenicity of *HCN2* haploinsufficiency.^43^

In conclusion, we confirm that pathogenic *HCN2* variants can be associated with more severe phenotypes than previously reported.^21^, ^22^, ^23^, ^24^ In addition, our findings highlight the association of mono and biallelic *HCN2* variants with ID with or without epilepsy. The epilepsy phenotypes in the individuals are variable, and most do not have features consistent with a named epilepsy syndrome. *HCN2* variants phenotypic spectrum also include dystonia and movement disorders, which have not been yet reported in individuals with *HCN2* or *HCN1* variants. Among 6 variants, only 1 leads to GoF, whereas 2 monoallelic variants induced partial LoF, or aberrant currents, and 3 biallelic variants caused complete LoF in *Xenopus* oocytes. Altogether, our findings also show that the impact of *HCN2* variants depends on the nature of defect, and their position in the HCN2 structure, but also on the genetic background and environmental factors. Finally, these in vitro functional data suggest a genotype–phenotype of *HCN2* variant correlation, suggesting that the greater the functional alteration induced by the *HCN2* variant, the more severe the pathology.

## Author Contributions

A.B., A.F.P., A.M.P., A.O.D.L., A.R., A.Z., B.D., B.K., C.E.M., C.L. (Claire Legendre), C.L. (Christian Legros), C.S., D.B., E.C., E.D., F.S.A., G.V.N., H.H., I.C.F., J.A.S., J.B., J.E.S., K.K., K.L.V.G., K.O., K.R., L.H.S., L.P., M.B., M.D., M.R., M.W., N.E., N.E.V., N.W.S., P.C., P.S., P.V.B., R.A.J., R.E.S., R.S.M., S.S., T.R., T.S., V.P., and V.S. contributed to the acquisition and analysis of data. A.M.P., C.H., C.A.R., C.L. (Christian Legros), D.B., D.H., G.L., M.C, K.B.H, and R.M. contributed to drafting the text or preparing the figures. A.M.P, A.Z., C.H., and C.L. (Christian Legros) contributed to the conception and design of the study.

## Potential Conflicts of Interest

The authors have no conflict of interest to declare.

## Supporting information


**Data S1.** Supporting Information
