# Supplementary material for: HCN2‐Associated Neurodevelopmental Disorders: Data from Patients and Xenopus Cell Models
Source: Ann Neurol. 2025 Jun 5;98(3):573–89. doi: 10.1002/ana.27277 (PMC12226820; doi:10.1002/ana.27277)
Supplement: Supplementary file 1 — Data S1. Supporting Information [file ANA-98-573-s001.pdf]

Characterization of HCN2-associated neurodevelopmental disorders using human and ranine models

Clara Houdayer, MSc, MD,[1†] A Marie Phillips, PhD,[2,3,†] Marie Chabbert, PhD,[4] Jennifer Bourreau, BS,[4] Reza Maroofian, PhD,[5] Henry Houlden, MD,[5] Kay Richards, PhD,[2] Nebal Waill Saadi, MD,[6] Eliška Dad'ová, MS,[4] Patrick Van Bogaert, MD, PhD,[7] Mailys Rupin, MD,[7] Boris Keren, MD,[8] Perrine Charles, MD, PhD,[8] Thomas Smol, MD, PhD,[9] Audrey Riquet, MD,[10] Lynn Pais, MS,[11,12] Anne O'Donnell-Luria, MD, PhD,[11,12] Grace E. VanNoy, MS,[11] Allan Bayat, MD, PhD,[13,14] Rikke S Møller, PhD,[13,14] Kern Olofsson, MD,[15] Rami Abou Jamra, MD,[16] Steffen Syrbe, MD, PhD,[17] Majed Dasouki, MD,[18] Laurie H Seaver, MD,[19,20] Jennifer A Sullivan, MS,[21],Vandana Shashi, MBBS, MD,[21] Fowzan S Alkuraya, MD,[22] Alexis F Poss, MS,[23] J Edward Spence, MD,[23] Rhonda E Schnur, MD,[24] Ian C Forster, PhD,[2] Chaseley E Mckenzie, MS,[2] Cas Simons, PhD,[25] Min Wang, PhD[25], Penny Snell, MGenCouns,[25] Kavitha Kothur, MD, PhD,[26] Michael Buckley, MD,[27], Tony Roscioli, MD, PhD,[27], Noha Elserafy, MD,[27] Benjamin Dauriat, MD,[28] Vincent Procaccio, MD, PhD,[1,29] Daniel Henrion, PhzrmD, PhD,[4] Guy Lenaers, PhD,[1,29] Estelle Colin, MD, PhD,[1,29] Nienke E. Verbeek, MD, PhD,[30] Koen L. Van Gassen, MD, PhD,[30] Claire Legendre, PhD,[4] Dominique Bonneau, MD, PhD,[1,29] Christopher A Reid, PhD,[2] Katherine B Howell, MBBS, PhD,[25,31] Alban Ziegler, MD, PhD,[1,29,32†] Christian Legros, PhD,[4†‡]

[†]These authors contributed equally to this work.

From the [1]Department of Medical Genetics, Angers University Hospital, 49933 Angers, France; [2]Florey Institute of Neuroscience and Mental Health, The University of Melbourne, Parkville Vic, Australia;[3]School of Biosciences, The University of Melbourne, Parkville, VIC, Australia; [4]Univ Angers, INSERM, CNRS, MITOVASC, Equipe CarMe, SFR ICAT, F-49000 Angers, France; [5]Department of Neuromuscular Disorders, UCL Queen Square Institute of Neurology, University College London, London, United Kingdom; [6]College of Medicine, University of Baghdad, Baghdad 10001, Iraq; Children Welfare Teaching Hospital, Medical City Complex, Baghdad 10001, Iraq; [7]Department of Pediatric Neurology, Angers University Hospital, 49933 Angers, France; [8]Department of Genetics, Pitié-Salpêtrière Hospital, Assistance Publique-Hôpitaux de Paris, 75019 Paris, Sorbonne University, France ; [9]Univ. Lille, CHU Lille, ULR7364 – RADEME, Institute of Medical Genetics, F-59000 Lille, France; [10]Department of Pediatric Neurology, Saint Vincent de Paul Hospital, GHICL, Lille, France; [11]Program in Medical and Population Genetics, Broad Institute of MIT and Harvard, Cambridge, MA, USA.

[12]Division of Genetics and Genomics, Boston Children's Hospital, Boston, MA, USA; [13]Department for genetics and personalized medicine, Danish Epilepsy Centre, Dianalund, Denmark; [14]Department Regional Health Research, University of Southern Denmark, Odense, Denmark; [15]Department of Paediatrics, Danish Epilepsy Centre Filadelfia, 4293 Dianalund, Denmark; [16]Institute of Human Genetics, University of Leipzig Medical Center, Germany; [17]Division of Paediatric Epileptology, Centre for Paediatrics and Adolescent Medicine, University Hospital Heidelberg, Heidelberg, Germany; [18]Department of Medical Genetics, Genomics and Personalized Health at AdventHealth-Orlando, Florida, USA; [19]Division of Medical Genetics, Corewell Health Helen DeVos Children's Hospital, Grand Rapids, MI, 49503, USA; [20]Department of Pediatrics and Human Development, Michigan State University College of Human Medicine, Grand Rapids, MI, 49503, USA; [21]Department of Pediatrics-Medical Genetics, Duke University, Durham, NC 27710, USA; [22]Department of Translational Genomics, Center for Genomic Medicine, King Faisal Specialist Hospital & Research Center, Riyadh, Saudi Arabia; [23]Pediatrics-Clinical Genetics and Metabolism, School of Medicine, University of Colorado-Anschutz Medical Campus, Aurora, Colorado, USA; [24]GeneDx, Gaithersburg, MD 20877, USA; [25]Murdoch Children's Research Institute, Melbourne, Australia; [26]Department of Neuropediatrics, The Children's Hospital at Westmead, Sydney Children's Hospital Network, Sydney, Australia; [27]New South Wales Health Pathology Randwick Genomics Laboratory, Sydney, NSW, Australia; [28]Department of Medical Genetics and Cytogenetics, Limoges University Hospital, France; [29]Univ Angers, INSERM, CNRS, MITOVASC, Equipe MitoLab, SFR ICAT, F-49000 Angers, France; [30]Department of Genetics, University Medical Center Utrecht, Utrecht, The Netherlands; [31]Department of Neurology, Royal Children's Hospital, Melbourne, Australia; [32]Department of Medical Genetics, University Hospital of Reims, Reims, France

[‡]Corresponding authors: Christian Legros

Corresponding author's address: Univ Angers, INSERM, CNRS, MITOVASC, Equipe CarMe, SFR ICAT, Bat. IRIS2, 3 rue Roger Amsler, F-4900, France

Corresponding author's phone : 00 33 244 688 273

Corresponding author's e-mail address: christian.legros@univ-angers.fr

Running head: Expanding *HCN2* variants phenotypic spectrum

Supplemental Material

Two-Electrode Voltage Clamp Recording in *Xenopus* oocytes

Adult female *Xenopus laevis* were purchased from CRB (Rennes, France) or TEFOR (Paris-Saclay, France) and bred in the animal facility in strict accordance with the recommendations of the Guide for the Care and Use of Laboratory Animals of the European Community (European Community council directive 2010/63/EU). Oocytes were harvested from mature female *Xenopus laevis* frogs under 0.15% tricaine anaesthesia. All animals recovered after 2-3 h. Every female is operated every three months, not less. A single female was used no more than 5 times. The experimenters followed the steps to avoid any animals' pain and suffering, and they understand the ethical principles and state that their work complies with its animal ethics checklist. No statistical test was realized to estimate the sample sizes. Only one measurement was performed with each oocyte. Injected oocytes were incubated 3-4 days at 18°C prior TEVC recordings. Electrophysiological recordings were replicated from 4 to 12 distinct oocyte collected from at least three different frogs. When Ih exceed 400 nA at -30 mV, or when oocytes were leaky, recordings were excluded. Randomization was achieved by random selection of frog or oocytes before injection.

TEVC recordings were conducted using a TEV-200 amplifier (Dagan Corporation, Minneapolis, USA). Digidata 1440A interface (Axon CNS Molecular Devices, San Jose, CA, USA) and pCLAMP™ 10.7 software (Axon CNS Molecular Devices) were used for current recording. Non-injected oocytes were used as negative controls and wt-HCN2 injected oocytes as positive controls. Injected oocytes were continuously perfused at room temperature with a standard oocyte saline solution (SOS) (in mM): 100 NaCl, 1 KCl, 1 $CaCl_2$, 1.8 $MgCl_2$, and 10 HEPES (pH = 7.5 using NaOH). Oocytes were impaled with electrodes filled with 0.5 M KCl/1.5 M K-acetate. A solution of $CsCl_2$ (5 mM in SOS) was used to block currents elicited by wt-HCN2 and its variants. For p.(Gly587Asp), p.(Pro493Leu) and p.(Leu377His) HCN2 variants, TEVC recordings were carried out in the Reid laboratory, Florey Institute of Neuroscience, Parkville, Australia using methods as described elsewhere.[1,2]

Currents were analysed using Clampfit 10.7 (Molecular Devices). Mean current amplitudes were calculated from at least five different cells from at least two different *Xenopus* females. Membrane capacitances were measured for each oocyte to determine current densities (nA/nF) which were used to analyse the current density-voltage relationship (I/V curves). I/V curves were plotted with steady-state and instantaneous currents. Slow currents were determined by subtracting instantaneous currents from steady-state currents. Current activation rates were analysed with classical exponential equation ($I = A.e^{-t/tau} + B$), where t corresponds to time, tau, the kinetics constant and B to the current when t = 0. For tail current or voltage-dependent activation analyses, we used the Boltzmann equation ($G = 1/(1 + \exp((V_{1/2} - V)/k))$), where V is the test potential, $V_{1/2}$ is the half-activation potentials and, k, the slope factor.

Western blot analysis

Western blotting was performed as previously described[3], with initial centrifugation steps added to remove yolk proteins. Samples of 15-20 oocytes, injected or un-injected, were collected, resuspended in 15 µl/lysis buffer {20 mM HEPES buffer (pH 7.4) with protease inhibitors (Complete, Boehringer Mannheim)}, and lysed by multiple passages through a fine syringe needle. Yolk proteins were removed from the lysates by a centrifugation at 1000 g for 10 min. The pellet was discarded, and supernatant protein levels analysed by the Bradford method (Bio-Rad CA, USA). Supernatants were stored at -80°C. Prior to electrophoresis the samples were thawed, and the buffer adjusted to 20 mM HEPES (pH = 7.4), 1% Triton X100, 0.1% sodium deoxycholate, 1% SDS, 140 mM sodium chloride, 2 M urea (final concentration). The homogenates were then incubated with rotation for 30 min at 4°C to solubilize membrane proteins before 1x reducing loading buffer was added and the samples incubated at 37°C for 1 h.[4] Thirty µg of protein from control and experimental samples were separated by electrophoresis on 8% SDS-PAGE gels, at 70-100 V, running buffer (Tris/glycine pH 8.3, 1% SDS) until the migration distance of 75 and 100 kDa size markers {(Pre-stained Precision Plus Dual Colour Standards (Bio-Rad, CA, USA)} reached approximately 3 cm. Proteins were transferred to nitrocellulose (Tris-glycine 1% SDS, 20% methanol), and visualized by Ponceau S staining (Sigma Aldrich,#. P3504). Blots were blocked in 0.5% skim milk TBSI, 1x TBS 0.5% IGEPAL CA-630, (Sigma Aldrich, #I8896) for 1 h at RT. before overnight incubation at 4°C, with blocking buffer containing 1:500 rabbit anti-HCN2 APC-030 (Alomone Labs Cat# APC-030, RRID: AB2313726). The filters were washed with TBSI thrice and incubated in blocking buffer with goat anti-rabbit Poly HRP 32260 (Thermo Fisher Scientific Cat# 32260, RRID:AB1965959) 1:15,000 for 1 h at RT. Beta- actin (Thermo Fisher Scientific Cat# PA1-183, RRID:AB_2539914) was used as a loading control. The protein signal was visualized with Clarity Western ECL Substrate (Bio-Rad, CA, USA) and the signal captured by a Bio-Rad ChemiDoc™ MP imaging system (Image Lab Software, RRID: SCR_014210).

Transient expression in HEK293 cells and confocal fluorescent microscopy

HEK293T cells were transfected with N-terminal EGFP tagged constructs encoding human wt-HCN2, and the biallelic variants, p.(Leu377His), p.(Pro493Leu) and p.(Gly587Asp). The EGFP tagged and truncated $HCN2_{\Delta C\text{-}X}$ (Thr553Ter) was used as negative control. The HCN2 constructs were tagged at their amino termini to avoid disruption of membrane trafficking.[5] All reagents used for cell culture and transfection were purchased from Thermo Fisher Scientific (Scoresby, Victoria, Australia) unless otherwise mentioned. Transfections were carried out using Invitrogen Lipofectamine 3000 in 24 well plates containing glass coverslips (13 mm diam.) coated with poly D-lysine hydrobromide (Merck KGaA, Darmstadt, Germany), according to the manufacturer's instructions. In brief, 24 hours after splitting 1x 10^5 cells/well, viability 99% were seeded in the medium, DMEM, high glucose (4.5 g/L), sodium pyruvate, no glutamine, supplemented with 10% GlutaMax Supplement, 10% fetal bovine serum, penicillin (100 U/ml) and streptomycin (100 mg) and cells were incubated for 24 hours, at 37°C and 5% CO_2. Exposure to light was minimized for all following steps. The cells were transfected with 0.5 µg HCN2 construct/ well, three wells per construct, and incubated for 24 hours. The medium was then replaced with medium containing CellMask Orange Plasma Membrane

Stain and the plate incubated at 37°C for 15 min, followed by fixing for 1 hour at 37°C in 4% Paraformaldehyde. The cells were washed thrice with PBS, stained with DAPI (1:2000 dilution) and washed again thrice with PBS. Coverslips with the stained cells were mounted on slides in *SlowFade* Gold Antifade Mountant solution, sealed with clear nail varnish and stored in the dark at 4°C for 18 hours before imaging on a Zeiss LSM900 (Florey Neuroscience Microscopy Facility). Over-view images were acquired using 20x/0.8 NA objective with three repeats per group. Higher resolution and images for analysis of relative sub-cellular expression pattern, membrane versus cytosol, was done using high-resolution 3D images acquired with Airyscan technique and C Plan-Apochromatic 63x/1.4 NA oil immersion objective (n=19-24 cells). Images were captured as 16 Bits, XY = 0.035 µm pixel size and Z step size=0.13 µm. For quantitative image analysis, all acquisition parameters were matched across all samples. The imaging parameters were cell mask orange for membrane (CMO) fluorescence signal (Em/Ex 556/572); EGFP-tagged constructs (Em/Ex 488/509) and DAPI nuclear stain (Em/Ex 353/465) were detected with a GaAsP-PMT detector type and wavelength detection ranges CMO:540-700; EGFP:490-545 and DAPI:400-487 respectively. Relative-quantification of GFP expression was done using Image- J.[6] Briefly, two ROIs for each cell, membrane and cytosol, were delineated. The membrane perimeter was defined using CMO and the remaining area assigned as cell cytosol. The mean GFP-signal intensity was then obtained for each ROI. The imager was "blind" to the genotype of the constructs.

Protein 3D modelling and structural analysis

MODELLER V9.17 was used to generate molecular models of depolarized and hyperpolarized HCN2-wt from position 163, by homology with the cryo-electron microscopy of human HCN1 (PDB 5U6P and 6UQF corresponding to depolarized and hyperpolarized models, respectively).[7,8] These templates were selected after comparison with rabbit HCN4 (PDB 7NMN).[9] HCN1 and HCN4 structures differ in the S4-S5 linker, with an Mg2+ binding site observed only in HCN4.[10] For each modelling, twenty structures were built and refined by simulated annealing (refine.fast option). The final models were selected according to the MODELLER PDFf score. The wt-HCN2 3D-models were subsequently used as templates for modeling the p.(His205Gln), p.(Arg324His), p.(Arg324Cys), p.(Ala363Val), p.(Asn369Ser), p.(Met374Leu), p.(Leu377His), p.(Gly460Asp), p.(Glu478del), p.(Pro493Leu) and p.(Gly587Asp) variants, in resting and hyperpolarized states. The 3D-models were graphically inspected with PyMOL (Molecular Graphics System, version 1.8, Schrödinger, LLC) to analyse the impact of each variant on HCN2 structure. The solvent accessible surface area (ASA) and the relative solvent accessibility (RSA) were calculated with PyMOL with a solvent radius of 1.4 Å. HCN2 structures were also analysed using two predictive and analytical tools developed by Biosig Lab

available at https://biosig.lab.uq.edu.au/. The tools called DDMut (https://biosig.lab.uq.edu.au/ddmut/) and DynaMut (https://biosig.lab.uq.edu.au/dynamut/) were used to compute the Gibbs free energy (ΔΔG) Δ vibrational entropy energy between wt-HCN2 and variant (ΔΔSVib ENCoM), giving information on structure flexibility.[11,12]

Supplementary Table 1 Genotypic and phenotypic characteristics of individuals carrying a pathogenic variant in HCN2, leading to a gain of function.

**Functional impact**	**Gain-of-function (n=18)**						
**Allelic status**	**Monoallelic (n=18)**						
Individual reference	**Individual 2**	**Individual 3**	**Individual 4**	**Dibbens, 2010[24] (n=6, unrelated)**	**Nakamura, 2013[26] (n=2, unrelated)**	**Li, 2018[27] (n=3, related)**	**Li, 2018[27] (n=4, 3 related, 1 unrelated)**
Variant information							
HCN2 variant	p.Arg(324His)			p.(Pro719-Pro721del)	p.(Ser126Leu)	p.(Val246Met)	p.(Ser632Trp)
Method of detection	whole exome sequencing	whole exome sequencing	whole exome sequencing	ND	ND	ND	ND
Inheritance	Paternal inherited	Maternal inherited	/	/			
Ion channel modification	Increases ion channel conductance			Increases ion channel conductance	Induces a positive shift of $V_{1/2}$ activation at high temperature only	Induce a positive shift of $V_{1/2}$ activation and faster current kinetics	
Demographics							
Age at last review (years)	9	/	/	/	/	/	/
Living? (Age at death if deceased)	Yes	/	/	/	/	/	/
Sex	F	M	F	/	F (2)	M (2), F (1)	M (2), F (2)
Family history	Migraines in maternal branch, social difficulties in father	Father of individual 2	Grandmother of individual 2	/	Affected mother and daughter	Affected mother and daughter	Affected relatives
Clinical features							
Development/cognition							
Diagnosis of DD/ID	Borderline	Borderline	Borderline	/	/	Yes (1/3)	No
DD/ID severity (IQ if available)	IQ 74	/	/	/	/	Mild (1/3)	/
Developmental plateau or regression?	Regression with seizures	/	/	/	/	/	/
Gross motor abilities	Ambulant	/	/	/	/	/	/
Language/communication abilities	Appropriate then regression	/	/	/	/	/	/
Behavioral/neuropsychiatric symptoms	ASD, ADHD	ASD, ADHD	Social difficulties	/	/	ADHD (1/3)	/
Epilepsy							
History of seizure	Yes	No	No	Yes 6/6	Yes 2/2	Yes (3/3)	Yes (4/4)
Diagnosis of epilepsy	Yes	NA	NA	Yes (1/6)	No	Yes (3/3)	Yes (3/4)
Age seizure onset	3yr	NA	NA	/	/	6yr (1), 10yr (1), adolescence (1)	3yr (1), / (3)
Seizure type(s)	Unclassified	NA	NA	FS (5), MA (1)	FS (2)	MS (2), TCS (3), possible focal seizure (1)	Absence (1), TCS (1), photosensitive seizures (2), FS (1)
EEG findings	SWAS	NA	NA	/	/	Generalized spike-wave	Generalized spike-wave
Epilepsy syndrome	Unclassified epilepsy that evolved into EE-SWAS	NA	NA	FS (3), GEFS+ (3 related individuals, who had MAE (1), FS+ (1) and FS (1), respectively)	/	JME (1), photosensitive IGE (1), unclassified (1)	IPOE (2), early-onset absence epilepsy (1), FS (1)
Ongoing seizures?	Seizure free	NA	NA	/	/	/	/
Antiseizure medication	Seizures controlled on ethosuximide, levetiracetam, sulthiame	NA	NA	/	/	/	/
Other neurological features							
Tone	Hypotonia	/	/	/	/	/	/
Movement disorder	No	/	/	/	/	/	/
Other clinical features							
Ophthalmologic features	No	Astigmatism	/	/	/	/	/
Dysmorphic features	Large mouth, full lips, long palpebral fissures, bulbous nose	/	/	/	/	/	/
Brain MRI features							
Description	Subcortical WM T2 hyperintensities	/	/	/	/	/	/

Abbreviation: Ab = absence seizures, ADHD = attention deficit hyperactivity disorder, ASD = autism spectrum disorder, ASM = antiseizure medication, BTCS = bilateral tonic-clonic seizures, CVI = cortical visual impairment, EE-SWAS = epileptic encephalopathy with spike-and-wave activation in sleep, F = female, FS = febrile seizure, GTCS = generalized tonic-clonic seizures, GCS = generalized clonic seizures, GEFS+ = genetic epilepsy with febrile seizures plus, GS = generalized seizures, ID = intellectual disability, IEDs = interictal

epileptiform discharges , IESS = infantile epileptic spasms syndrome, IGE = idiopathic generalized epilepsy, IQ= intellectual quotient, IPOE = idiopathic photosensitive occipital lobe epilepsy, JME = juvenile myoclonic medication, M = male, MAE = myoclonic astatic epilepsy, MS = myoclonic seizures, NA = not applicable, ND = not done, SE = status epilepticus, SWAS = spike-and-wave activation in sleep, TCS = tonic-clonic seizure, WM = white matter, yr = years, / = unknown.

Supplementary Table 2 Genotypic and phenotypic characteristics of individuals carrying a pathogenic variant in HCN2, leading to a loss of function.

**Functional impact**	**Loss-of-function (n=12)**											
**Allelic status**	**Monoallelic (n=5)**					**Biallelic (n=7)**						
Individual reference	**Individual 6**	**Individual 7**	**Individual 9**	**DiFrancesco, 2023[28] (n=1)**	**Individual 12**	**Individual 10**	**Individual 11**	**Individual 16**	**Individual 17**	**Individual 18**	**Individual 19**	**DiFrancesco, 2011[25] (n=1)**
Variant information												
HCN2 variant	p.(Ala363Val)		p.(Met374Leu)	p.(Gly460Asp)		p.(Leu377His)		p.(Pro493Leu)			p.(Gly587Asp)	p.(Glu515Lys)
Method of detection	ND	ND	Ex. sequencing	ND	Ex. sequencing	Ex. sequencing		Genome sequencing			Ex. sequencing	ND
Inheritance	De novo	/	De novo	De novo	De novo	Parental		Parental			Parental	Parental
Ion channel modification	Lead to the absence of current despite protein expression			Lead to a reduction of current due to trafficking defect[28]		Lead to the absence of current despite protein expression, loss of membrane trafficking						Induces a negative shift of $V_{1/2}$ activation
Demographics												
Age at last review (years)	44	61	8	11	2	8	5	13yr 8mo	11	8yr 2mo	6	/
Living? (Age at death if deceased)	No (49 yr)	Yes	Yes	Yes	Yes	Yes	Yes	Yes	Yes	Yes	No (8 yr)	/
Sex	M	F	F	F	F	M	M	F	M	F	M	M
Family history	No	Yes	No	No	No	No		No			No	No
Development/cognition												
Diagnosis of DD/ID	Yes	Yes	Yes	Yes	Yes	Yes	Yes	Yes	Yes	Yes	Yes	Not stated
DD/ID severity (IQ noted where available)	Moderate (IQ42)	Mild (IQ60)	Borderline (IQ71) → Moderate (IQ54)	Moderate (IQ40)	Moderate	Severe	Severe	Severe	Severe	Severe	Severe to profound	/
Developmental plateau or regression?	No	No	Regression	No	No	No	No	No	No	No	Regression	/
**Gross motor abilities**<br>**Brain MRI features**<br>Description	Walk (20 mo)	/	Walk (18 mo)	Delayed	Stands with support, unable to walk	Sit (2-3 yr), unable to walk	Sit (23 mo), unable to walk	Unable to sit unaided	Sit (5 yr)	Walk (7yr)	Unable to walk (4 yr)	/
Language/communication abilities	Delayed	Delayed	Delayed	Severely impaired	Single words	Nonverbal	Nonverbal	Nonverbal	Nonverbal	Nonverbal	Nonverbal (4yr)	/
Behavioral/neuropsychiatric symptoms	No	/	No	/	CVI, limited pointing and response to name	No	No	Limited eye contact	Limited eye contact	No	CVI	/
Epilepsy												
History of seizure	Yes	No	Yes	Yes	Yes (FS only)	No	Yes	No	Yes	No	Yes	Yes
Diagnosis of epilepsy	Yes	No	Yes	Yes	No	No	Yes	No	Yes	No	Yes	Yes
Age seizure onset	9 mo	NA	11 mo	5mo	6mo	NA	2.5 mo	NA	3 yr	NA	4 mo	12 yr
Seizure type(s)	Clonic, then TCS seizures	NA	FS then focal seizures (likely Rolandic)	SE then GS	Single FS	NA	Infantile spasms	NA	GCS, Ab	NA	Focal seizures	Ab, TCS, possibly MS
EEG findings	Focal abnormality (right temporo-parietal)	NA	Focal central sharp wave	Asynchronous bilateral posterior epileptic discharges	NA	NA	EEG at presentation not available, at 9mo background slow	NA	/	NA	Multifocal IEDs, well organized background apart from absence of sleep spindles on first two EEGs	Generalized sharp-wave complexes
Epilepsy syndrome	Unclassified	NA	Unclassified	Dravet syndrome	NA	NA	IESS	NA	Unclassified	NA	Unclassified	Generalized epilepsy (syndrome not specified)
Ongoing seizures?	/	NA	/	SE	No	NA	Seizure free since 8mo (off ASM at 18mo)	NA	/	NA	Seizure free since 2 yr 3 mo (off ASM age 4y)	/
Antiseizure medication	Phenobarbital, clobazam,	NA	Levetiracetam (response not stated)	Drug-resistant seizures	NA	NA	Steroids, topiramate and	NA	Clonazepam and levetiraceta	NA	Seizures controlled with levetiracetam,	Levetiracetam (response not stated), previous treatments: valproate (incompletely effective), lamotrigine

	lamotrigine (response not stated)			reduced on 1.5:1 ketogenic diet			vigabatrin (response not stated)		m (response not stated)		resistant to clonazepam and topiramate	(initial benefit), topiramate (incompletely effective)
Other neurological features												
Tone	Pyramidal signs	Pyramidal signs predominant in the lower limbs	Normal	Dystonic-spastic tetraparesis	Hypotonia	Hyperreflexia	Hypotonia	Hypertonia	Hypertonia	Axial hypotonia, appendicular hypertonia	Hypotonia	/
Movement disorder	Yes (not detailed)	Static cerebellar signs	No	Cerebellar signs with tremor, dysmetria and ataxia	No	Hand stereotypies	No	Dystonia, hand stereotypies	Dystonia	Dystonia, hand stereotypies, tremor	Stereotypies	/
Other clinical features												
Ophthalmologic features	Ocular albinism (biallelic TYR variants)	Nystagmus	No	Vertical and horizontal nystagmus	Upgazed nystagmus	Exotropia	Nystagmus, hyperopic astigmatism, intermittent exotropia	No	No	Nystagmus	Nystagmus	/
Dysmorphic features	No	5th finger clinodactyly	No	/	Low set years, flat philtrum, brachycephaly	No	No	Sparse hair, curved nails, overlapping toes, widely spaced nipples	Overlapping toes, widely spaced nipples)	Sparse hair, curved nails, widely spaced nipples	Deep set eyes	/
Miscellaneous	Died from pancreatic cancer	Cervical and lumbar spinal stenosis	Microcephaly, numbness and pain in legs, VUR	/	Gastroesophageal reflux, laryngomalacia	Coxa valga, hypopigmented macule, capillary malformations	Coxa valga	Bruxism, constipation	Bruxism, constipation	Poor sleep	High pain tolerance, poor sleep, recurrent vomiting	/
Brain MRI features												
Description	Atrophy	Atrophy, few subcortical WM T2 hyperintensities	Normal	Normal	ND	Normal	Normal	ND	ND	ND	Dysmorphic corpus callosum, unusual sulcation	Normal

Abbreviation: Ab = absence seizures, ADHD = attention deficit hyperactivity disorder, ASD = autism spectrum disorder, ASM = antiseizure medication, BTCS = bilateral tonic-clonic seizures, CVI = cortical visual impairment, EE-SWAS = epileptic encephalopathy with spike-and-wave activation in sleep, Ex sequencing = Exome sequencing, F = female, FS = febrile seizure, GTCS = generalized tonic-clonic seizures, GCS = generalized clonic seizures, GEFS+ = genetic epilepsy with febrile seizures plus, GS = generalized seizures, ID = intellectual disability, IEDs = interictal epileptiform discharges , IESS = infantile epileptic spasms syndrome, IGE = idiopathic generalized epilepsy, IQ= intellectual quotient, IPOE = idiopathic photosensitive occipital lobe epilepsy, JME = juvenile myoclonic medication, M = male, MAE = myoclonic astatic epilepsy, mo = month, MS = myoclonic seizures, NA = not applicable, ND = not done, SE = status epilepticus, SWAS = spike-and-wave activation in sleep, TCS = tonic-clonic seizure, WM = white matter, yr = years, / = unknown.

Supplementary Table 3 Genotypic and phenotypic characteristics of individuals carrying a pathogenic variant in HCN2 with unknown functional impact.

**Functional impact**	**Unknown functional impact (n=8)**							
**Allelic status**	**Monoallelic (n=6)**						**Biallelic (n=2)**	
**Individual reference**	**5**	**8**	**13**	**14**	**15**	**1**	**20***	**21***
Variant information								
HCN2 variant	p.(Arg324Cys)	p.(Asn369Ser)	p.(Glu478del)			p.(His205Gln) // p.(Ser409Leu)	p.(Met647HisfsTer31)	
Method of detection	Ex. sequencing		Ex. sequencing		Ge. sequencing	Ge. sequencing		
Inheritance	De novo	De novo	De novo	De novo	De novo	Maternally inherited // De novo	Parental	
Ion channel modification	Unknown							
Demographics								
Age at last review (years)	13	42	11	4	4 yr 9 mo	2	10	8
Living? (Age at death if deceased)	Yes	Yes	Yes	Yes	Yes	Yes	Yes	Yes
Sex	M	M	F	M	F	F	M	F
Family history	Father with learning difficulties/Mother with ADHD	No	No	No	No	Infantile spasms in maternal branch (second- and third-degree relatives)	No	
Development/cognition								
Diagnosis of DD/ID	No	Yes	Yes	Yes	Yes	Yes	Yes	Yes
DD/ID severity (IQ noted where available)	IQ107	Mild	Mild	Severe	Unknown	Severe	Severe	Severe
Developmental plateau or regression?	Mild regression around 10 years	UKN	No	No	No	No	No	No
**Gross motor abilities** **Brain MRI features** Description	Normal	UKN	Sit (24 mo), unable to walk	Unable to sit unaided	Stands but unable to walk	Unable to walk	Unable to walk	Walk (4 yr)
Language/communication abilities	Normal	Needed speech therapy	Few words, Makaton, able to read	No words, vocalizes	No words, vocalizes	Singles words	Nonverbal	Single words
Behavioral / neuropsychiatric symptoms	ADHD, autistic features (no formal ASD diagnosis)	Hospitalized with episode of delirium at age 35 years	No	CVI	Limited eye contact	CVI	No	No
Epilepsy								
History of seizure	Yes	Yes	No	No	Yes (FS only)	No	Yes	Yes
Diagnosis of epilepsy	Yes	Yes	No	No	No	No	Yes	Yes
Age seizure onset	5yr	6yr	NA	NA	/	NA	Infancy	1 mo
Seizure type(s)	Focal seizure (Rolandic) → BTCS	staring spells, TCS	NA	NA	Single FS	NA	GTCS	Tonic eye deviation
EEG findings	Focal IEDs (centrotemporal)	Generalized and multifocal IEDs	Normal	NA	NA	Normal	Multifocal IEDs, slow background	IEDs (spikes), intermittent focal background slowing
Epilepsy syndrome	Focal epilepsy	Unclassified	NA	NA	NA	NA	Unclassified	Unclassified
Ongoing seizures?	Yes (~1 every 3 months)	Yes	NA	NA	NA	NA	Yes	Seizure free
Antiseizure medication	Clobazam and lamotrigine currently, previously oxcarbazepine (side effects), valproate (ineffective)	Perampanel, oxcarbazepine, zonisamide currently (response not stated). Previous treatments: valproate (response not stated), vagus nerve stimulation (no benefit) callosotomy (response not stated)	NA	NA	NA	NA	Levetiracetam, topiramate, clobazam (partially controlled)	Seizures controlled with levetiracetam
Other neurological features								
Tone	Normal	No	Axial hypotonia, spastic paraparesis	Hypotonia in infancy, progressive spastic paraparesis	Spasticity	Hypotonia	Axial hypotonia, lower limb spasticity	Hypotonia
Movement disorder	No	No	Dystonia, tremor	No	Dystonia, stereotypies	Dystonia, Sandifer syndrome	No	No
Other clinical features								
Ophthalmologic features	No	/	Nystagmus, severe myopia, astigmatism and pale papillae	Nystagmus	Nystagmus, optic nerve hypoplasia, strabismus	No	No	No

Dysmorphic features	No	No	No	No	No	Deep set eyes, epicanthal folds, micrognathia, plagiocephaly	No	No
Miscellaneous	4 café-au-lait spots	Obesity	Dysarthria, drooling	Bruxism, drooling	No	Dysautonomia, gastroesophageal reflux, torticollis, neonatal hepatitis	No	Bullous skin lesions
Brain MRI features								
Description	Mild asymmetry temporal horns	Mild T2 signal change in periventricular WM posteriorly	Periventricular WM abnormalities, vermis hypoplasia/atrophy	T2 hyperintensity in WM (bilateral parietal and occipital, cerebellar), and in thalamus, basal ganglia, pons, dentate nuclei	T2 hyperintensity in WM (periventricular), and in thalamus	Normal	Normal	Normal

Abbreviation: Ab = absence seizures, ADHD = attention deficit hyperactivity disorder, ASD = autism spectrum disorder, ASM = antiseizure medication, BTCS = bilateral tonic-clonic seizures, CVI = cortical visual impairment, EE-SWAS = epileptic encephalopathy with spike-and-wave activation in sleep, F = female, FS = febrile seizure, GTCS = generalized tonic-clonic seizures, GCS = generalized clonic seizures, GEFS+ = genetic epilepsy with febrile seizures plus, GS = generalized seizures, ID = intellectual disability, IEDs = interictal epileptiform discharges , IESS = infantile epileptic spasms syndrome, IGE = idiopathic generalized epilepsy, IQ = intellectual quotient, IPOE = idiopathic photosensitive occipital lobe epilepsy, JME = juvenile myoclonic medication, M = male, MAE = myoclonic astatic epilepsy, mo = months, MS = myoclonic seizures, NA = not applicable, ND = not done, SE = status epilepticus, SWAS = spike-and-wave activation in sleep, TCS = tonic-clonic seizure, WM = white matter, yr = years, / = unknown, // = trans-variants.

Supplementary Table 4 Genomic findings of *HCN2* variants.

Genomic coordinates (GRCh37/hg19)	HGVS cDNA NM_031304.5	HGVS Protein	Exon numbering	GnomAD Population Allele Frequencies (NFE/Total) & (Homozygote?)	Computational Prediction Scores				Zygosity	ACMG Classification
					CADD	PROVEAN	Mutation Assessor	DANN		
Chr19:590560C>A	c.615C>A	p.(His205Gln)	1/8	Not present	24.6	Damaging (-6,02)	Medium (3,225)	0.9879	Cpd het	Likely Pathogenic (PM2, PM3, PP3, PP4)
Chr19:603882G>A	c.971G>A	p.(Arg324His)	2/8	Not present	25.8	Damaging (-4,34)	Medium (3,42)	0.9993	Het	Pathogenic (PS3, PM2,PP3 PM5)
Chr19:603881C>T	c.970C>T	p.(Arg324Cys)	2/8	Not present	26.1	Damaging (-6.94)	Pathogenic (3.42)	0.999	Het	Likely Pathogenic (PS2, PM2, PM5, PP3)
Chr19:605092C>T	c.1088C>T	p.(Ala363Val)	3/8	Not present	23.1	Damaging (-3,36)	Medium (2,34)	0.9989	Het	Likely Pathogenic

										(PS2, PS3, PM1, PM2, PP3)
Chr19:605110A>G	c.1106A>G	p.(Asn369Ser)	3/8	Not present	22.5	Damaging (-4.37)	Low (1,905)	0.9957	Het	Likely Pathogenic (PS2, PM1, PM2, PP3)
Chr19:605124A>C	c.1120A>C	p.(Met374Leu)	3/8	Not present	23.4	Damaging (-2,62)	Low (1,635)	0.957	Het	Pathogenic (PS2, PS3, PM1, PM2, PP3)
Chr19:605134T>A	c.1130T>A	p.(Leu377His)	3/8	Not present	27.0	Damaging (-5,99)	Medium (3,395)	0.9845	Hom	Pathogenic (PS3, PM1, PM2, PP3)
Chr19:607971C>T	c.1226C>T	p.(Ser409Leu)	4/8	One occurrence in NFE	23.3	Damaging (-4,75)	Medium (2,19)	0.9989	Cpd het	Likely Pathogenic (PM2, PM3, PP3, PP4)
Chr19:608124G>A	c.1379G>A	p.(Gly460Asp)	4/8	Not present	NA	Damaging (-6,71)	Pathogenic (3,675)	0,995	Het	Pathogenic (PS2, PM1, PM2, PP3)

Chr19:608175delAGG	c.1432_1434del	p.(Glu478del)	4/8	Not present	NA	NA	NA	NA	Het	Likely Pathogenic (PS2, PM1, PM2, PP3)
Chr19:610299C>T	c.1478C>T	p.(Pro493Leu)	5/8	Not present	NA	Damaging (-9,7)	Medium (3,005)	0.9984	Hom	Likely Pathogenic (PS3, PM2, PP3)
Chr19:613423G>A	c.1760G>A	p.(Gly587Asp)	6/8	One occurrence in NFE	27.0	Damaging (-6.78)	Pathogenic (4.685)	0.9986	Hom	Pathogenic (PS3, PM2, PP3)
Chr19:613963insT	c.1936_1937insT	p.(Met647HisfsTer32)	7/8	Not present	NA	NA	NA	NA	Hom	Likely Pathogenic (PVS1, PM2, PM3)

Abbreviations: ACMG, American college of medical genetics; Cmp Het, Compound Heterozygous; Het, Heterozygous; HGVS, human genome variation society; Hom, Homozygous; gnomAD, genome aggregation database; NFE, non-Finnish European; NA, not available.

Supplementary Table 5 Structural impact analysis of HCN2 variants.

	Depolarized state			Hyperpolarized state			
	**ΔΔG (kcal/mol)**	**SASA (Å²) WT**	**variant**	**ΔΔG (kcal/mol)**	**SASA (Å²) WT**	**variant**	**Destabilizing (yes/No)**
**HCN2 variants**							
p.(His205Gln)// p.(Ser409Leu)	-2.85	3.07<br>69.85	6.96<br>115.45	-1.9	2.78<br>62.75	0<br>113.15	Yes
p.(Arg324His)	1.09	139.27	100.50	0,05	24.07	14.12	No
p.(Arg324Cys)	0,63	139.27	88.88	0,76	24.07	9.44	No
p.(Ala363Val)	0,72	11.60	6.67	0,13	17.13	32.26	No
p.(Ans369Ser)	1.63	43.49	36.26	0	77.59	50.99	No
p.(Met374Leu)	0.36	25.59	36.42	1,11	28.86	31.69	No
p.(Leu377His)	-0,85	53.20	56.59	-0,26	57.03	80.84	Yes
p.(Gly460Asp)	-0,12	3.07	0	-0,12	0	1.28	Yes
p.(Pro493Leu)	-2.76	57.99	107.21	-0,22	54.92	89.14	Yes
p.(Gly587Asp)	-3.42	11.35	14.99	-3,87	3.07	3.82	Yes

ΔΔG values were determined using DDMut predictor. SASA = Solvent Accessibility Surface Area of the corresponding residue was calculated with PyMol. ΔΔG<0 indicates destabilizing effects, while ΔΔG>0 predicts stabilizing effects.

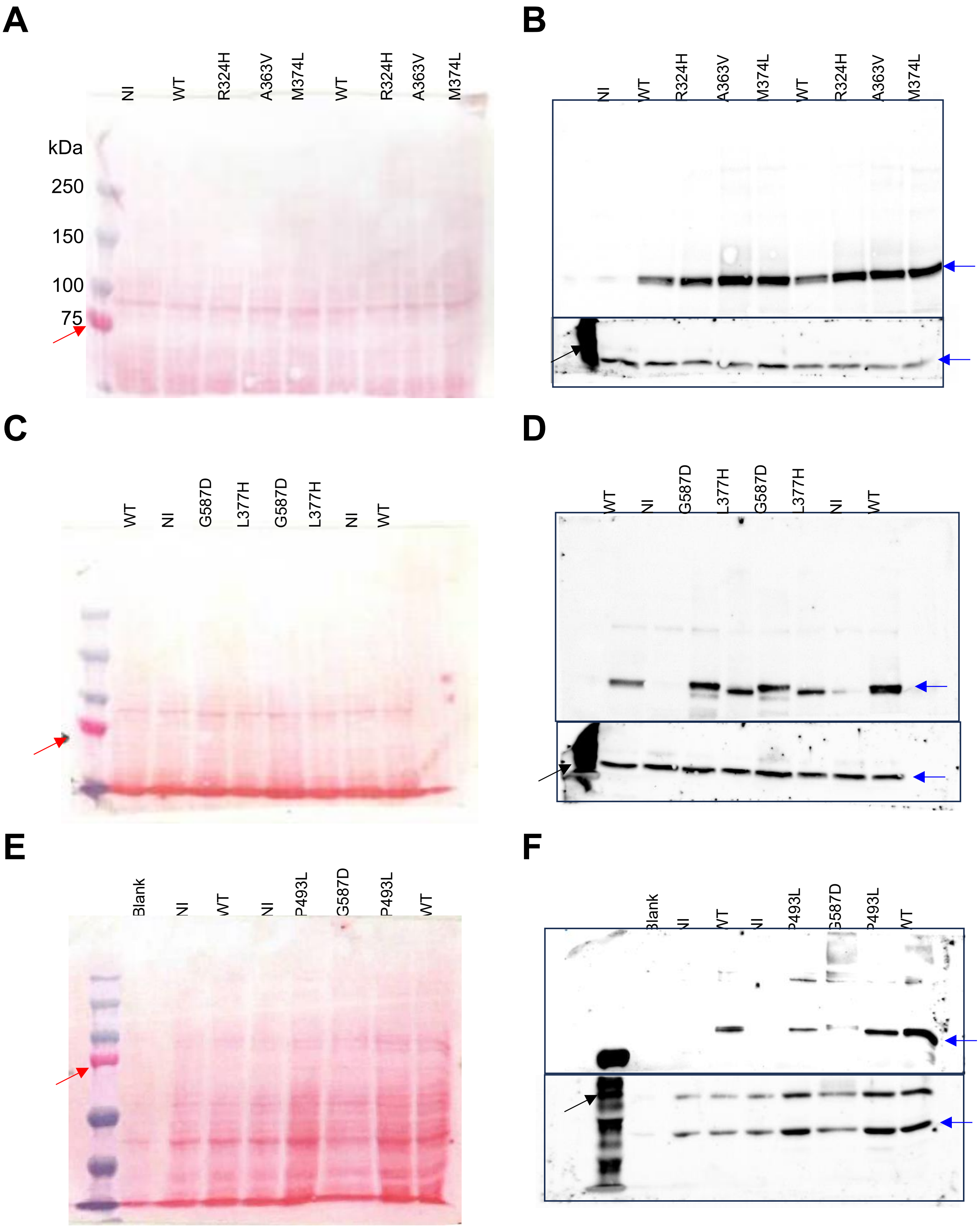

Supplementary Figure 1 Western blot analysis of wt-HCN2 and its variants identified in this study. Thirty µg of protein extracts from oocytes expressing wt-HCN2 and its variants were analyzed by immunoblotting. (A,C,E) Ponceau stained blot of protein extracts from non-injected oocytes (NI), wt-HCN2 controls (WT) and HCN2 variants (R324H, A363V, M374L, L377H, P493L and G587D). Red arrow indicates the position blot was cut prior to addition of primary antibodies. (B,D,F) Immunostained blots with anti-HCN2 (upper box) and anti-β-actin antibodies (lower box). Blue arrows show cross-reacting protein with ~100 kDa for HCN2 and 42 kDa for β-actin. The slightly smaller L377H and P493L protein is assumed to result from a post-translational difference as no molecular reason could be detected by sequencing. The dark mark on the left is due to cross-reactivity with the size markers (black arrows).

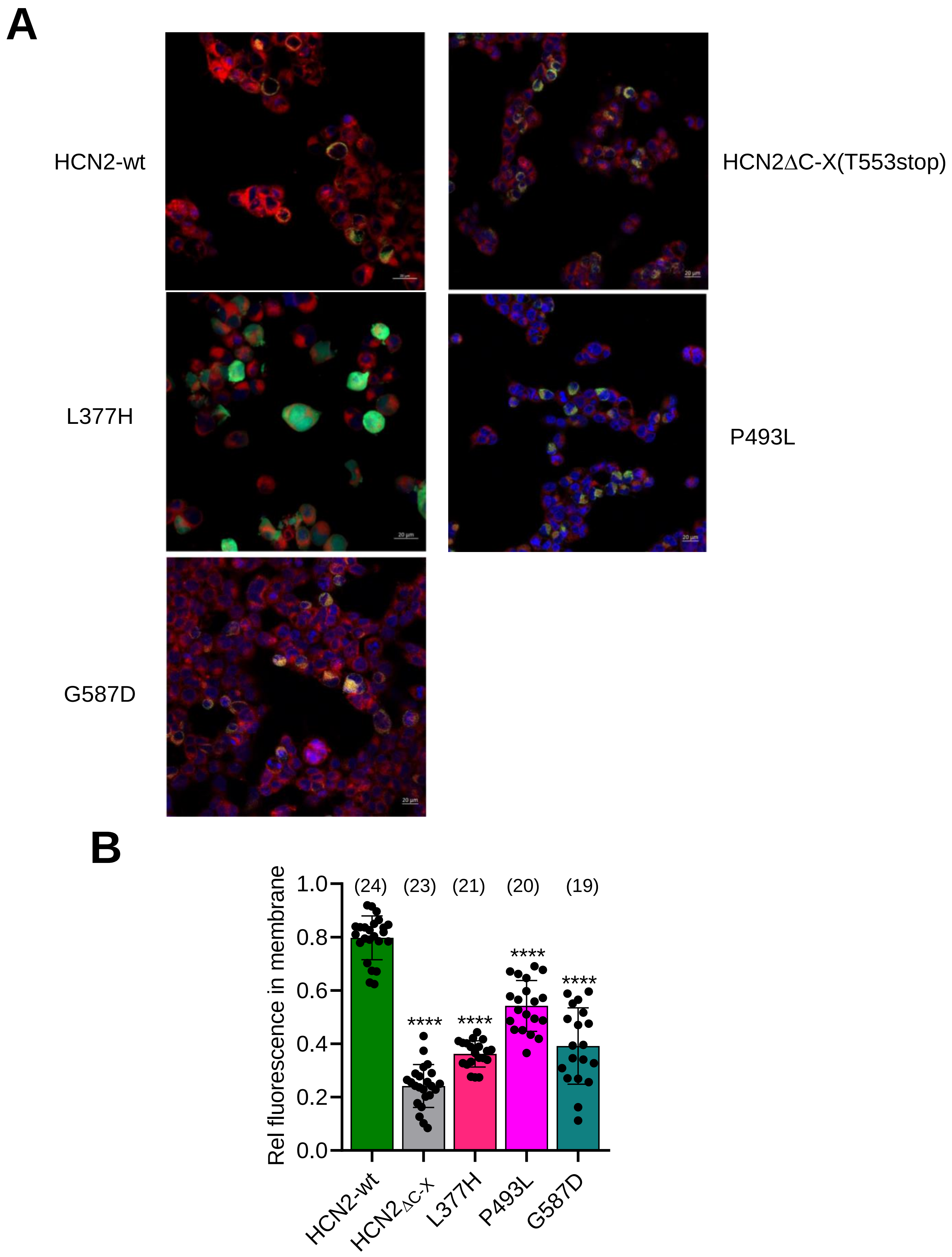

Supplementary Figure 2 Membrane trafficking of p.(Leu377His), p.(Pro493L) and p.(Gly587asp) HCN2 variants in HEK293 cells. (A) The cellular localization of the expression of EGFP-tagged p.(Leu377His), p.(Pro493L) and p.(Gly587asp) HCN2 variants was investigated by confocal microscopy. CellMask orange and DAPI were used to stain the membrane and nuclei, respectively. The images shown here were acquired with magnification from 60x. **(A)** Relative-quantification of GFP expression in the membrane was evaluated using Image-J as described above. The numbers of cells analyzed are shown in brackets. Data are mean±SD, n=19-24 cells. Data were analyzed by One way ANOVA test followed by a Dunnett's multiple comparison test. Means of relative fluorescence in membrane obtained for each construction were compared with that of HCN2-wt (****: p<0.0001).

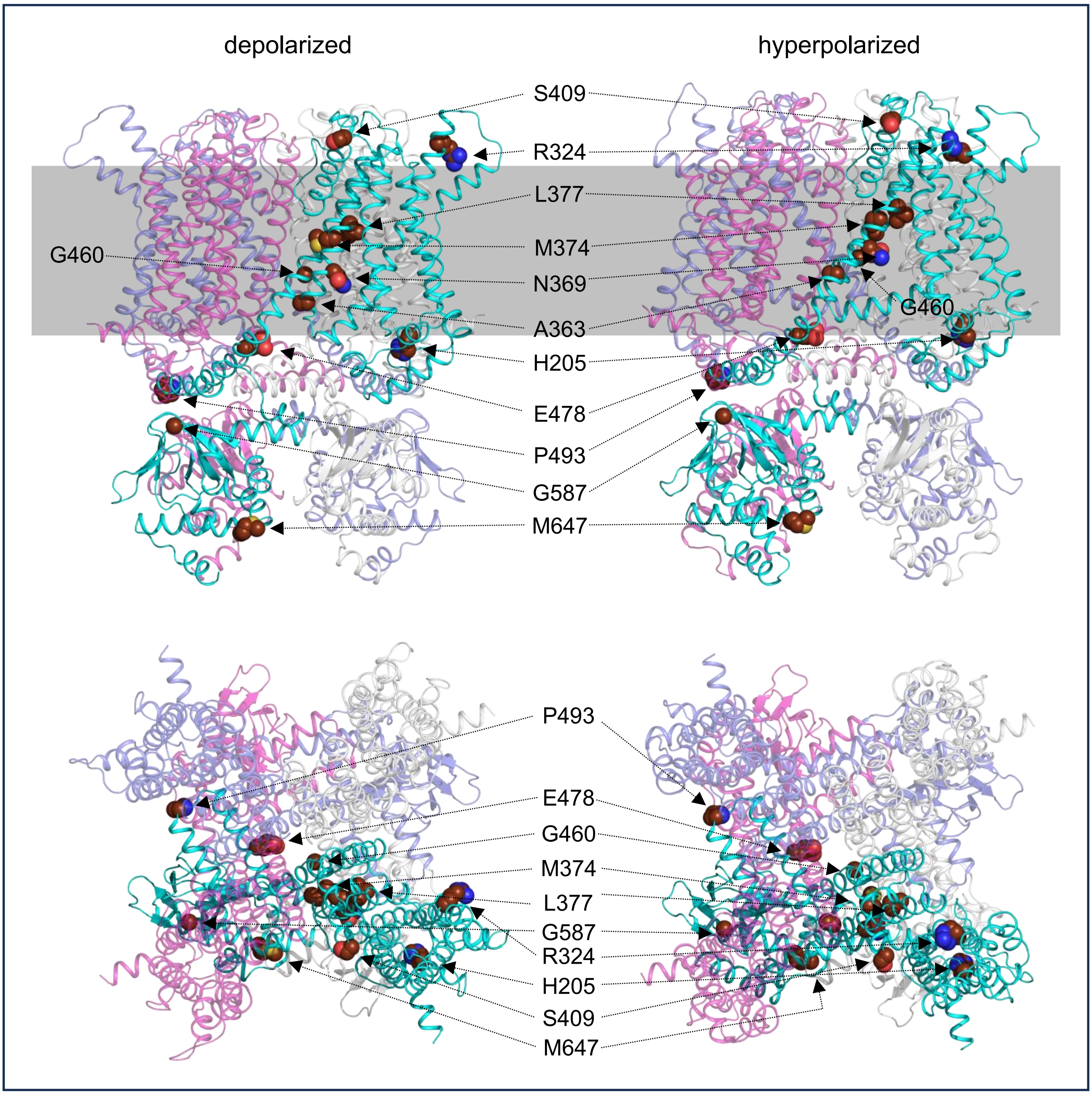

Supplementary Figure 3 Mutation mapping onto 3-dimensional homology models of HCN2 in depolarised and hyperpolarised states. Models of HCN2 in depolarized (left panels) and hyperpolarized states (right panels) are shown (upper panels: profile views. lower panels: top views). Proteins are drawn in ribbon representation with a different color code for each subunit. For clarity, the locations of mutated amino acids are represented as spheres in only one subunit (in cyan), while the other subunits are shown in semi-transparent representations. The approximate position of HCN2 within the membrane is visualized with the gray rectangle (profile views).

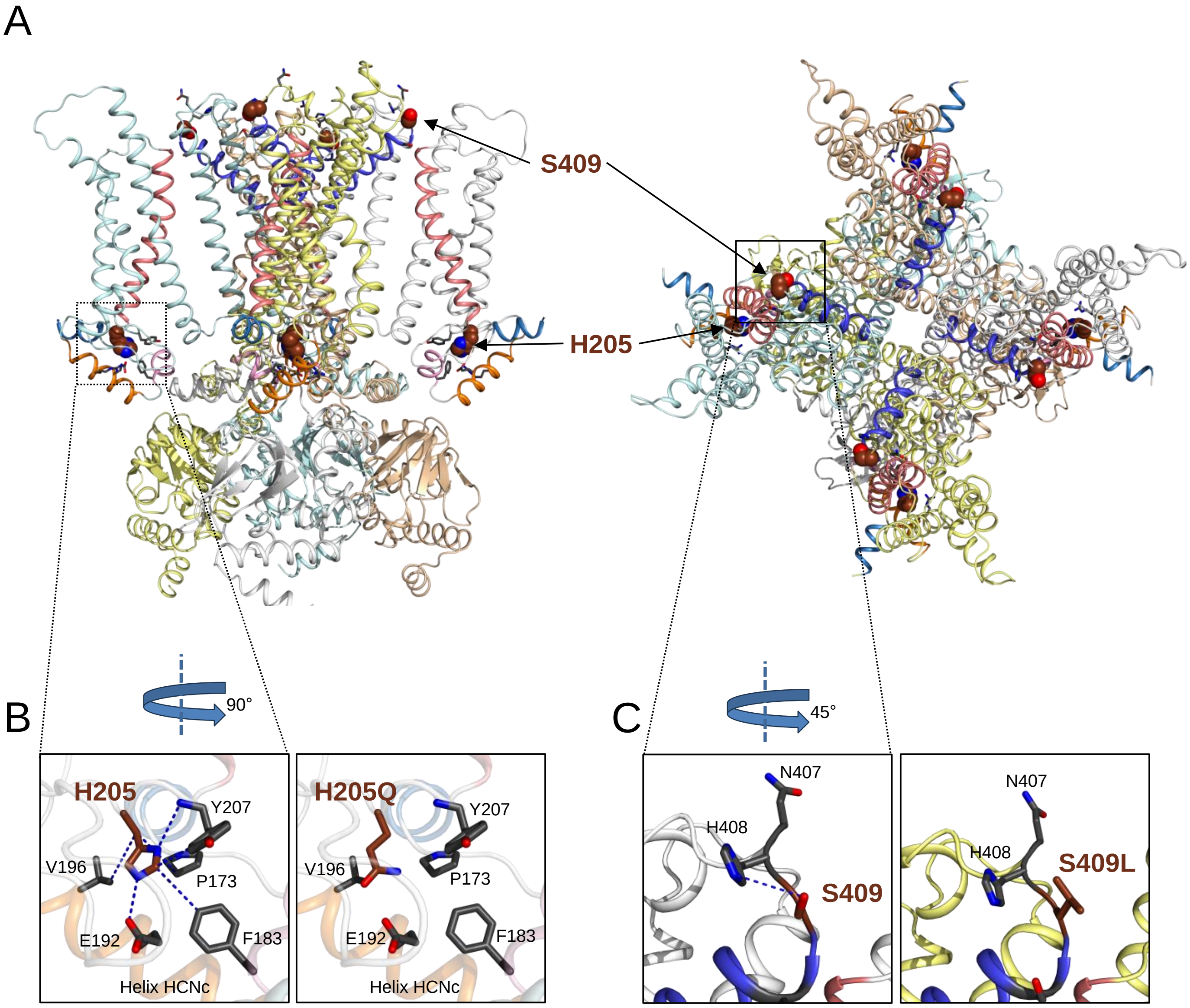

Supplementary Figure 4 Structural analysis of p.(His205Gln)/p.(Ser409Leu) trans variant. A) Top (left) and profile (right) views of HCN2 3D structure in depolarized state. His205 (spheres) is located at the N-terminal part of S1 and its side-chain is buried in a pocket formed by Pro173 (helix HCNa in light blue), Phe183 (helix HCNb in pink), Glu192 and Val196 (helix HCNc in orange) and Tyr207 (S1 in salmon). The side-chain of Ser409 faces the extracellular side close to Ser411. (**B,C**) The 3D structure of p.(His205Gln)/p.(Ser409Leu) trans variant was modelized V9.17 with MODELLER. **B)** Detail view of His205 environment in depolarized HCN2-wt (profile view). The side-chain of His205 (left panel) is highly stabilized by three interaction types : 1) polar interactions, a salt bridge with Glu192 and a hydrogen bond with Tyr207, 2) aromatic interactions with Phe183 and Tyr207, and 3) hydrophobic interactions with Pro173 and Ala196. The p.(His205Gln) variant (right panel) leads to the neutralization of a buried charge (His205), the disruption of 1) polar interactions with Glu192 and Tyr207, and 2) aromatic interactions with Phe183 and Tyr207. **C)** In depolarized state, the side-chain of S409 makes a hydrogen bond with His408, which is lost in p.(Ser409Leu) variant. Altogether, p.(His205Gln)/p.(Ser409Leu) trans HCN2 variant impact HCN2 structure stability.

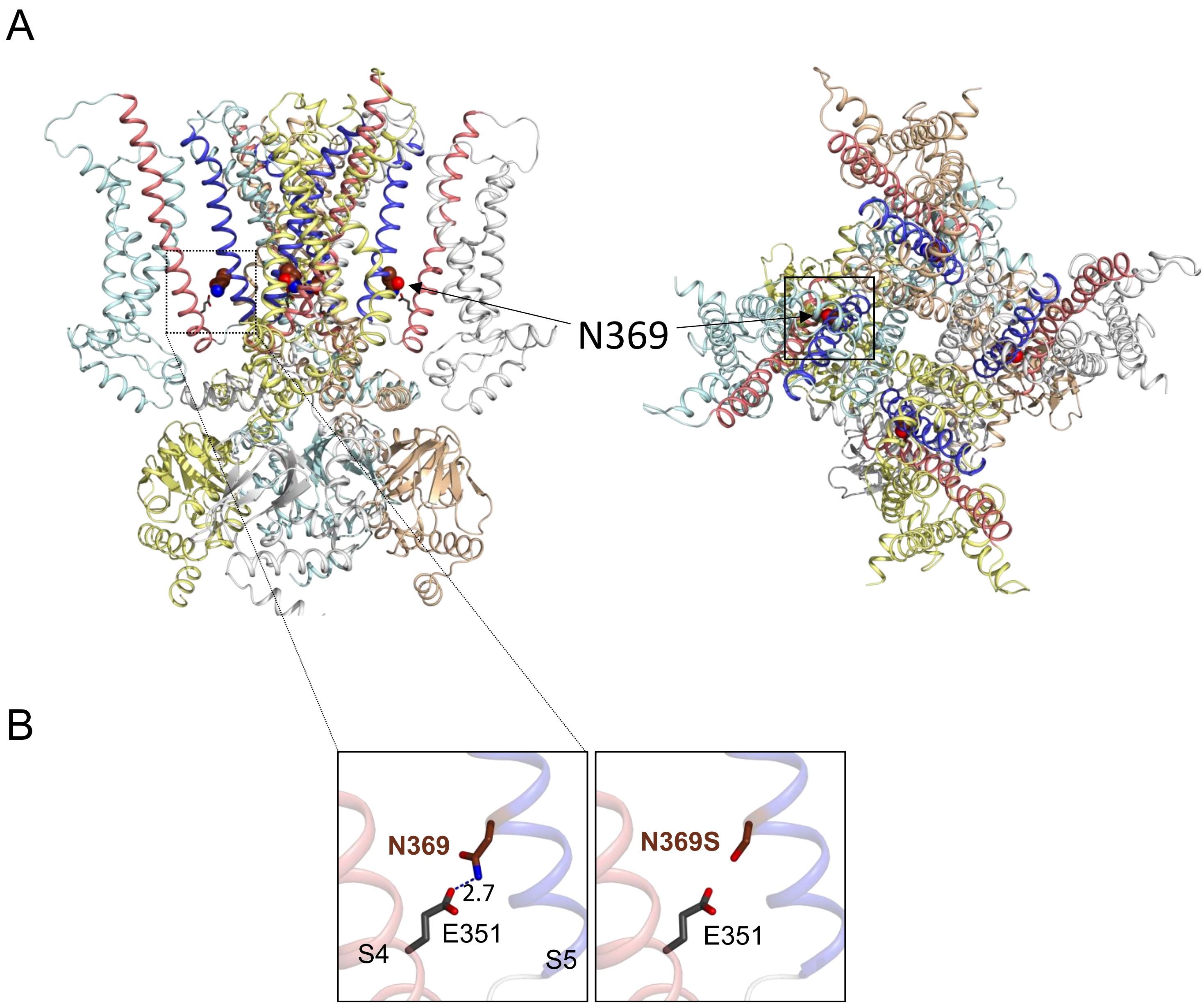

Supplementary Figure 5 Structural analysis of p.(Asn369Ser) pathogenic variant. A) Top (left) and profile (right) views of HCN2 3D structure in depolarized state. Asn369 (spheres) is located at the N-terminal part of S5 (in blue), and its side-chain faces S4 (in pink). (B,C) The 3D structure of p.(Asn369Ser) monoallelic variant was modelized V9.17 with MODELLER. B) Detail view of Asn369 environment in depolarized HCN2-wt (profile view). The side-chain of Asn369 (left panel) a hydrogen bond with Glu351. The p.(Asn369Ser) variant (right panel) leads to the disruption of the interaction with Glu351, and thus destabilizes the interface S4-S6. The distances are in Å.

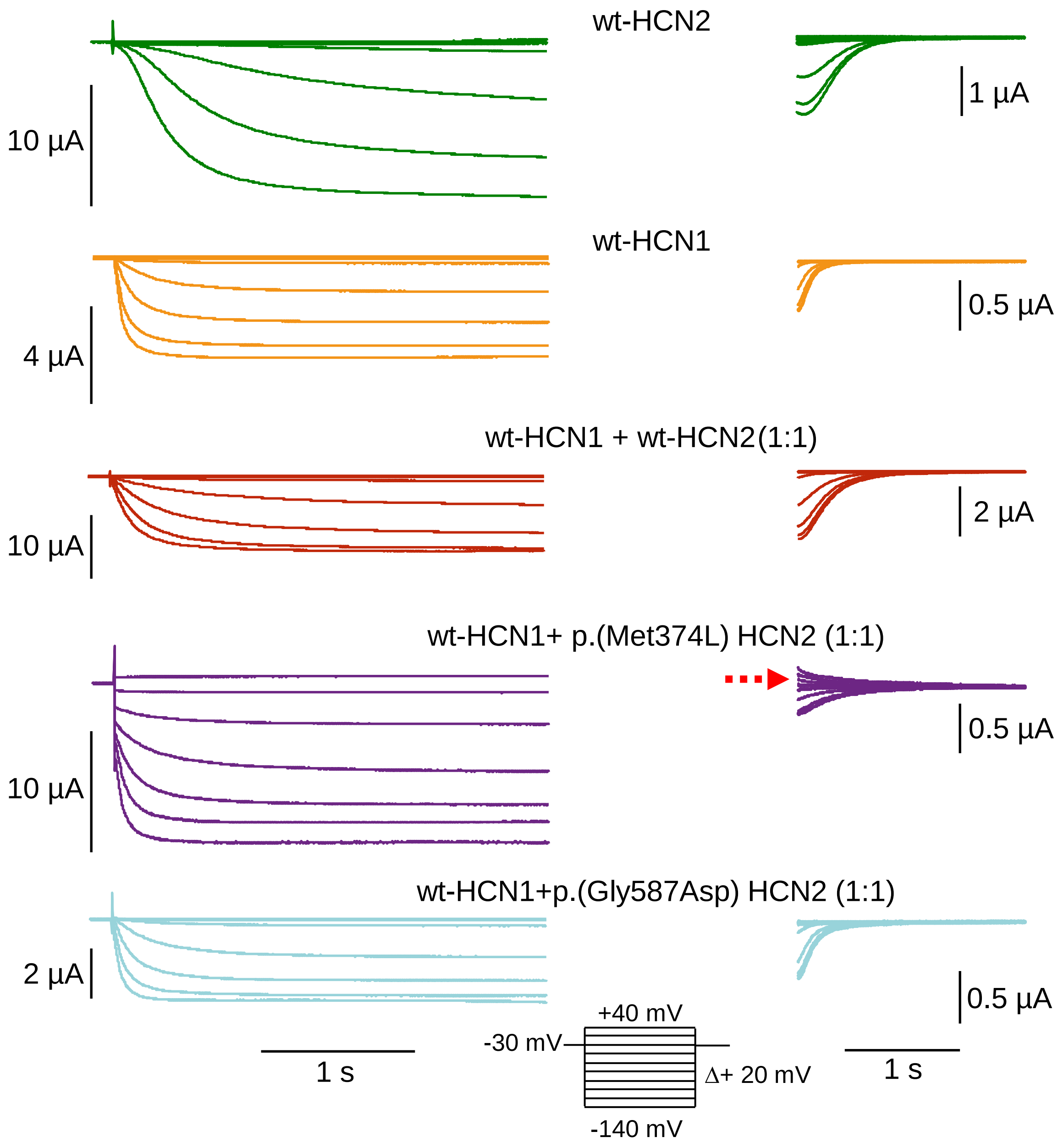

Supplementary Figure 6 Co-expression of p.(Met374Leu) and p.(Gly587Asp) HCN2 variants with wt-HCN1 channels in in *Xenopus* oocytes. From top to bottom, examples of superimposed current traces recorded in *Xenopus* oocytes, expressing wt-HCN2, wt-HCN1, wt-HCN1/wt-HCN2, wt-HCN1/p.(Met374Leu) HCN2, and wt-HCN1/p.(Gly587Asp) HCN2. The Cs-subtracted activation and the tails currents are shown on the left and right, respectively. Co-expression of heterotetrameric HCN1/HCN2 channels were performed by injection in *Xenopus* oocyte of their respective RNAs mixed in a 1:1 ratio. Only wt-HCN1/p.(Met374Leu) HCN2 heterotetramers showed outward tails (red dashed arrow). All experiments were performed as described elsewhere.[1,2]

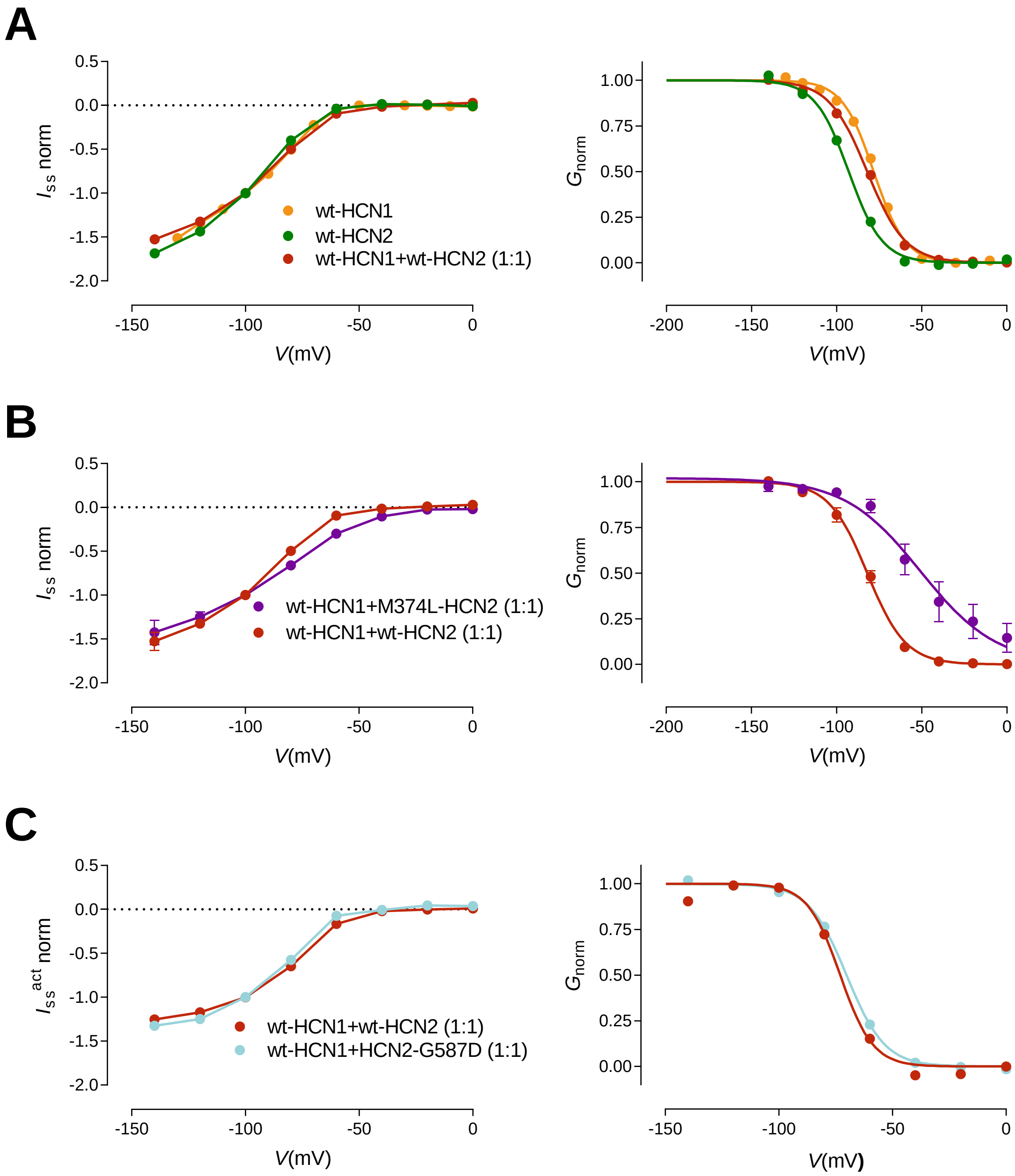

Supplementary Figure 7 Effects of p.(Met374Leu) and p.(Gly587Asp) HCN2 variants on HCN1/HCN2 heterotetramers in *Xenopus* oocytes. Co-expression of heterotetrameric HCN1/HCN2 channels were performed by injection in *Xenopus* oocyte of their respective RNAs mixed in a 1:1 ratio. The curves on the left, illustrating the voltage-dependency of steady-state activated currents (I_{ss}^{act} norm) by hyperpolarizing steps were superimposed for (A) wt-HCN2, wt-HCN1 and wt-HCN1/wt-HCN2 heterotetramers, (B) wt-HCN1/wt-HCN2 and wt-HCN1/p.(Met374Leu) HCN2 heterotetramers and (C) wt-HCN1/wt-HCN2, and wt-HCN1/p.(Gly587Asp) HCN2 heterotetramers. After Cs-subtraction, the currents were normalized with the currents at -100 mV. The curves on the right show the normalized conductance (G_{norm}) obtained from tail currents at -30 mV. The data were fitted with the Boltzmann equation: wt-HCN1 (V_{50} = -78.2 ± 0.4 mV, n = 3), wt-HCN2 (V_{50} = -92.9 ± 1.9 mV, n=3), wt-HCN1/wt-HCN2 (V_{50} = -81.9 ± 0.8 mV, n = 6), wt-HCN1/p.(Met374Leu) HCN2 (V_{50} = -50.8 ± 4.6 mV, n=4), and wt-HCN1/p.(Gly587Asp) HCN2 (V_{50} = -70.2±1.5 mV, n = 3). Data are mean±SEM. These data showed that the p.(Met374Leu) HCN2 variant co-assembled with wt-HCN1 to form a leaky channel, similarly to what reported for the p.(Met05Leu) HCN1 variant.[1,2]

References

1. Hung A, Forster IC, Mckenzie CE, et al. Biophysical analysis of an HCN1 epilepsy variant suggests a critical role for S5 helix Met-305 in voltage sensor to pore domain coupling. Prog. Biophys. Mol. Biol. 2021;166:156–172.

2. McKenzie CE, Forster IC, Soh MS, et al. Cation leak: a common functional defect causing *HCN1* developmental and epileptic encephalopathy. Brain Commun. 2023;5(3):fcad156.

3. Kharouf Q, Phillips AM, Bleakley LE, et al. The hyperpolarization-activated cyclic nucleotide-gated 4 channel as a potential anti-seizure drug target. Br. J. Pharmacol. 2020;177(16):3712–3729.

4. Laemmli UK. Cleavage of Structural Proteins during the Assembly of the Head of Bacteriophage T4. Nature 1970;227(5259):680–685.

5. Santoro B, Wainger BJ, Siegelbaum SA. Regulation of HCN Channel Surface Expression by a Novel C-Terminal Protein-Protein Interaction. J. Neurosci. 2004;24(47):10750–10762.

6. Schindelin J, Arganda-Carreras I, Frise E, et al. Fiji: an open-source platform for biological-image analysis. Nat. Methods 2012;9(7):676–682.

7. Sali A, Blundell TL. Comparative protein modelling by satisfaction of spatial restraints. J. Mol. Biol. 1993;234(3):779–815.

8. Lee C-H, MacKinnon R. Structures of the human HCN1 hyperpolarization-activated channel. Cell 2017;168(1–2):111-120.e11.

9. Saponaro A, Bauer D, Giese MH, et al. Gating movements and ion permeation in HCN4 pacemaker channels. Mol. Cell 2021;81(14):2929-2943.e6.

10. Saponaro A, Thiel G, Moroni A. Structural and functional approaches to studying cAMP regulation of HCN channels. Biochem. Soc. Trans. 2021;49(6):2573–2579.

11. Zhou Y, Pan Q, Pires DEV, et al. DDMut: predicting effects of mutations on protein stability using deep learning. Nucleic Acids Res. 2023;51(W1):W122–W128.

12. Rodrigues CH, Pires DE, Ascher DB. DynaMut: predicting the impact of mutations on protein conformation, flexibility and stability. Nucleic Acids Res. 2018;46(W1):W350–W355.
